# A multiomics analysis identifies retinol metabolism in fibroblasts as a key pathway in wound healing

**DOI:** 10.1172/jci.insight.194188

**Published:** 2025-10-16

**Authors:** Till Wüstemann, Elizabeta Madzharova, Mateusz S. Wietecha, Norbert B. Ghyselinck, Marcus Höring, Gerhard Liebisch, Nicola Zamboni, Ulrich auf dem Keller, Sabine Werner

**Affiliations:** 1 Institute of Molecular Health Sciences, Department of Biology, ETH Zurich, Zurich, Switzerland.; 2 Department of Biotechnology and Biomedicine, Technical University of Denmark, Kgs. Lyngby, Denmark.; 3 Department of Oral Biology, College of Dentistry, University of Illinois Chicago, Chicago, Illinois, USA.; 4 Institut de Génétique et de Biologie Moléculaire et Cellulaire (IGBMC), Département de Génétique Fonctionnelle et Cancer, Centre National de la Recherche Scientifique (CNRS UMR7104), Institut National de la Santé et de la Recherche Médicale (INSERM U1258), Université de Strasbourg (UNISTRA), Illkirch Cedex, France.; 5 Institute of Clinical Chemistry and Laboratory Medicine, University of Regensburg, Regensburg, Germany.; 6 Institute of Molecular Systems Biology, Department of Biology, ETH Zurich, Zurich, Switzerland.

**Keywords:** Cell biology, Dermatology, Metabolism, Metabolomics, Proteomics, Skin

## Abstract

Impaired wound healing poses a major and increasingly frequent health problem. Among the key players in the healing process are fibroblasts, but their metabolic profile in healing wounds is largely unknown. Using a combination of transcriptomics, targeted proteomics, and metabolomics, we identified retinol metabolism as a top regulated pathway in wound fibroblasts. This is functionally relevant, since even a mild retinol deficiency caused a delay in wound closure and reepithelialization, which mainly resulted from misdirected keratinocyte migration on the new granulation tissue. Quantitative proteomics identified integrin subunit α11 as a less abundant protein in wounds of mice subjected to a retinol-deficient diet. Reduced levels of this fibroblast-specific protein likely altered the granulation tissue matrix, which in turn affected reepithelialization. These results provide a comprehensive overview of the transcriptome, proteome, and metabolome of wound fibroblasts and identify retinol metabolism in fibroblasts as a key regulator of tissue repair.

## Abstract 2

A multi-omics approach combined with functional studies in mouse models identified retinol metabolism as a key pathway in wound repair

## Graphical abstract

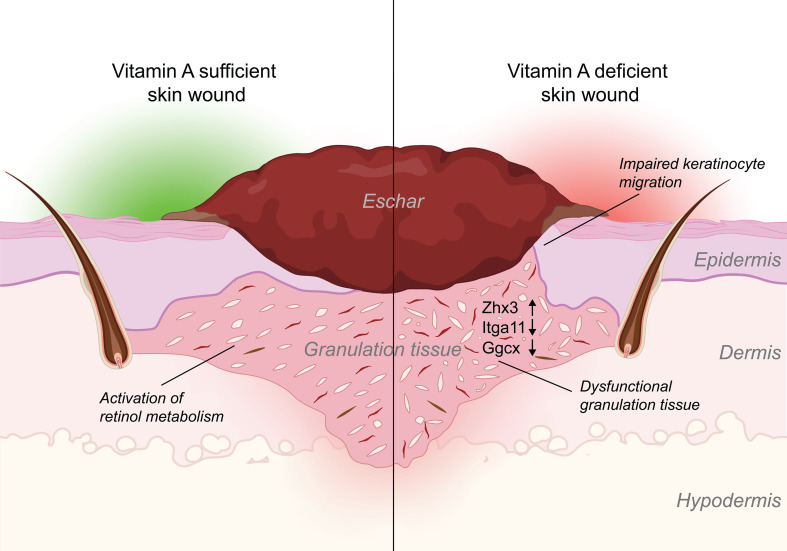

## Introduction

Wound healing requires the coordinated action of different cell types to achieve the restoration of tissue integrity (1). It involves 3 partially overlapping phases, including blood clotting and inflammation, new tissue formation, and tissue remodeling (2). One of the key cell types involved in skin wound healing are fibroblasts. During the proliferative phase of wound healing, they migrate into the wound area, proliferate, and deposit the majority of extracellular matrix (ECM) proteins. This allows keratinocytes at the wound edge to migrate on top of the newly formed granulation tissue and to seal the wound with a new epidermis, which reestablishes the important skin barrier. While recent studies have focused on cell fate, expression profiles, and spatial distribution of fibroblasts in skin wounds (3–9), much less is known regarding their metabolic features. A drastically reshaped environment, which wounds pose, heavily alters cellular metabolism and challenges the cells in the wound tissue to optimally allocate resources and metabolites (10, 11). Previous studies identified the necessity of functional bioenergetic pathways for successful wound healing and highlighted the importance of glycolysis in macrophages for efficient healing (12, 13). In addition, distinct metabolic profiles were detected for wound tissue compared with normal skin (14). In fibrotic skin, metabolic changes in fibroblasts altered their ECM deposition and arrangement (15). However, the metabolome of fibroblasts during wound healing has not been analyzed in an unbiased way, though these cells regulate their surrounding ECM depending on their metabolic state (15).

To fill this knowledge gap, we characterized the fibroblast metabolome of normal mouse skin and of wounds during the phase of granulation tissue formation and compared it with their transcriptome. Together with targeted and quantitative proteomics data of normal and wounded skin and functional wound healing studies, these data identify retinol metabolism as a key pathway in wound healing.

## Results

### Wounding activates the retinol pathway in fibroblasts.

**Figure 1:**
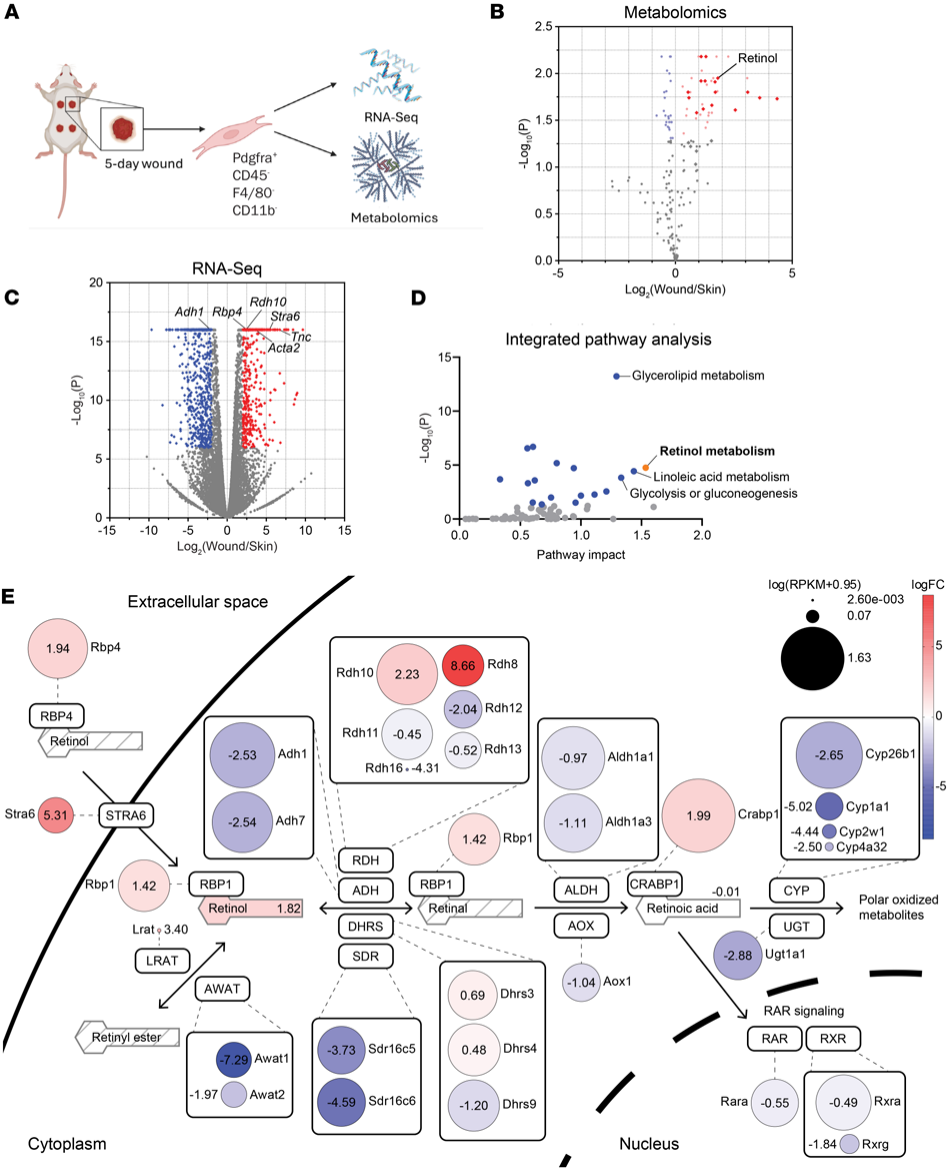
Retinol metabolism is strongly regulated by skin wounding. (**A**) Outline of the experimental setup. Pdgfrα^+^ fibroblasts were sorted from normal skin and 5-day wounds and subjected to RNA-Seq and metabolomics analysis. *N* = 3 mice per experiment. Created in BioRender. Werner, S. (2025) https://BioRender.com/aaw9vrp (**B**) Volcano plot showing differentially abundant metabolites in wound vs. normal skin fibroblasts. Colored points indicate metabolites with significantly differential abundance (*P* ≤ 0.05); diamond-shaped points indicate metabolites that were confirmed in an independent experiment. *N* = 3 mice per experiment. (**C**) Volcano plot showing differentially expressed genes in wound vs. normal skin fibroblasts based on bulk RNA-Seq analysis of FACS-sorted fibroblasts (16). Colored points indicate significantly differentially expressed genes (Bonferroni-corrected *P* ≤ 0.05, |log_2_(FC)| ≥ 2). *N* = 3 mice. *Rdh10*, retinol dehydrogenase 10; *Rbp4*, retinol-binding protein 4; *Stra6*, stimulated by retinoic acid 6. (**D**) Integrated pathway analysis of RNA-Seq and metabolomics data sets using MetaboAnalyst 6.0. Colored points indicate significantly altered pathways (*P* ≤ 0.05). (**E**) Schematic overview of retinol metabolism with highly relevant metabolites and genes. Metabolites with a strikethrough pattern were not detected in the metabolomics experiment. Members of the respective enzyme families that were significantly differentially expressed based on RNA-Seq data are featured (uncorrected *P* ≤ 0.05). Size of the circles corresponds to the log_10_(RPKM+0.95) (0.95 was added to all values to avoid negative results of the logarithm), and color represents the log_2_(fold change) of the genes (red: increased expression in wound vs. normal skin fibroblasts; blue: reduced expression in wound vs. normal skin fibroblasts). RPKM, reads per kilobase million.

To identify changes in the fibroblast metabolome and transcriptome that occur upon wounding, we FACS-sorted fibroblasts from nonwounded skin and 5-day full-thickness excisional wounds of wild-type mice as described before (16) using antibodies against the pan-fibroblast marker platelet-derived growth factor receptor α (Pdgfrα; CD140a), which is not expressed by dermal endothelial cells (4, 17). The 5-day time point was chosen, because it represents the peak of the phase of new tissue formation (18). We gated for live CD45^–^F4/80^–^CD11b^–^ nonimmune cells to exclude immune cells that occasionally express Pdgfra (19) and then for Pdgfrα^+^ cells. Cell lysates were subjected to untargeted metabolomics analysis (Figure 1A). Skin and wound tissue were treated identically to exclude alterations in metabolites that result from the tissue dissociation and sorting procedure (20). We also reanalyzed our previously published RNA-Seq data from skin and 5-day wound fibroblasts of mice of the same genetic background for genes that are regulated by wounding in wild-type mice (Figure 1A) (16).

Twenty-one unique metabolites were differentially abundant between normal and wounded skin and detected in 2 independent wound healing experiments (Figure 1B). RNA-Seq data confirmed the efficient separation of fibroblasts from endothelial cells as revealed by the low levels of the endothelial-specific Pecam1, Kdr, Cdh5, Cldn5, and Vwf transcripts (Supplemental Figure 1A; supplemental material available online with this article; https://doi.org/10.1172/jci.insight.194188DS1). There were strong alterations in the fibroblast transcriptome upon wounding (Figure 1C). Gene ontology overrepresentation analysis of the RNA-Seq data identified multiple pathways involved in ECM constitution and binding that were significantly overrepresented in wound fibroblasts (Supplemental Figure 1B).

The identical preparation of fibroblasts for the metabolomics (this study) and transcriptomic analyses (16) allowed us to integrate these data sets and to determine how the expression of metabolism-associated genes matches with the metabolites present in the cell. There was a strong correlation between the wound-enriched metabolites and mRNAs encoding the enzymes required for their production or proteins involved in their transport. We focused on the significance of the alteration and the pathway impact, which captures the relevance of a pathway. Among the significantly altered pathways, retinol (vitamin A) metabolism scored highest on pathway impact (Figure 1D).

Analysis of retinol metabolism genes and metabolites within the acquired data revealed strong alterations in multiple components of this pathway (Figure 1E and Supplemental Table 1). Genes that are more highly expressed in wound versus normal skin fibroblasts encode proteins involved in storage and trafficking of retinoids, such as retinol-binding protein 4 (Rbp4), which is required for the release from its stores in the liver and transport in the serum, the retinol receptor and transporter stimulated by retinoic acid 6 (Stra6), which promotes uptake of retinol by cells (21), and the intracellular Rbp1, which is responsible for retinol transport to acyltransferases for storage as retinol esters or to dehydrogenases for stepwise oxidization to retinoic acid (RA) (22–24). Similarly, *Crabp1*, which encodes the cellular RA-binding protein 1, was expressed at higher levels in wound fibroblasts. This intracellular RA chaperone is responsible for RA transfer into the nucleus to induce gene expression changes via binding to RA receptor (RAR)/retinoid X receptor heterodimers (Figure 1E).

The most strongly regulated step in the retinol pathway was the oxidation of retinol. Several genes of this rate-defining step of retinol metabolism (25) were strongly regulated upon wounding, e.g., retinol dehydrogenases 8 and 10 (*Rdh8* and *Rdh10*; upregulated) and short-chain dehydrogenase/reductase family 16C members 5 and 6 (*Sdr16c5* and *Sdr16c6*; downregulated). Expression of most of the enzymes responsible for the oxidation of retinal to RA was downregulated, in particular aldehyde dehydrogenase 1 family member A1 (*Aldh1a1*). These findings suggest enhanced uptake of retinol by wound fibroblasts, its accumulation in these cells, and a concomitant reduction in the expression of genes associated with retinol metabolization to RA. This reduction may be a compensatory mechanism to maintain appropriate levels of RA, which activates RAR-mediated gene expression.

Together, these results identify retinol metabolism as a key pathway that is activated in wound fibroblasts. Analysis of published single-cell RNA-Seq (scRNA-Seq) data from mouse excisional wounds at different stages (26) confirmed this finding and also revealed that this pathway is mainly activated between day 3 and 7 after wounding, especially in fibroblasts (Supplemental Figure 1, C and D).

### Retinol metabolism genes are activated by retinoids in cultured fibroblasts.

**Figure 2:**
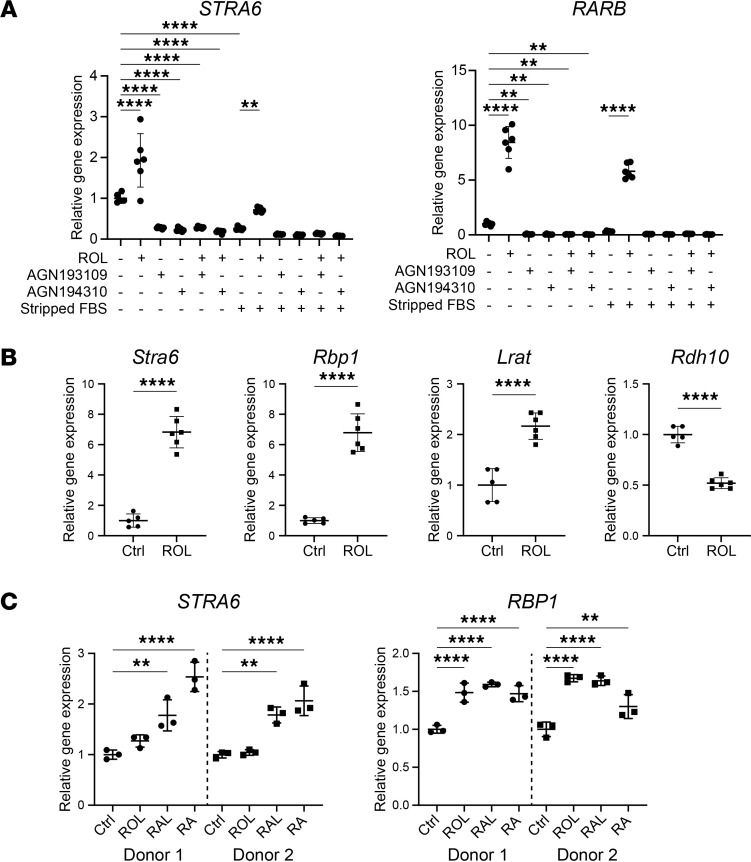
Retinol metabolism genes are regulated by retinoids in cultured fibroblasts. (**A**) Real-time quantitative PCR (RT-qPCR) for *STRA6* and *RARB* relative to *RPL27* using RNA from primary human fibroblasts, treated with 1 μM retinol (ROL), 0.1 μM pan-RAR inverse agonist (AGN193109), 0.1 μM pan-RAR inhibitor (AGN194310), charcoal-stripped FBS (Stripped FBS), or untreated FBS (for samples without Stripped FBS treatment) for 24 hours. *N* = 6 cultures from 1 donor per treatment group. (**B**) RT-qPCR for *Stra6*, *Rbp1*, *Lrat*, and *Rdh10* relative to *Rps29* using RNA from primary mouse fibroblasts, treated with 1 μM ROL for 24 hours after 48 hours of starvation in medium supplemented with 1% FBS. *N* = 5–6 cultures from 2–3 mice per treatment group. (**C**) RT-qPCR for *STRA6* and *RBP1* using RNA from primary human fibroblasts from 2 different donors (1 and 2), treated with 1 μM ROL, retinal (RAL), or RA for 24 hours after 48 hours of starvation in serum-free medium. *N* = 3 cultures per donor and treatment group. Graphs show mean ± SD. **P < 0.01, ****P < 0.0001, 1-way ANOVA, Šídák’s multiple comparisons test (**A** and **C**), or Mann-Whitney test (**B**).

We next searched for potential regulators of retinol metabolism in the wound environment. Because wound fibroblasts are exposed to serum, which induces a wound fibroblast phenotype (27), and because serum contains high levels of retinol (in mice and humans around 1 μmol/L) (28, 29), we tested the effect of serum and of retinol itself on the expression of retinol metabolism genes. We focused on *STRA6*/*Stra6*, *RBP1*/*Rbp1*, *RPB4*/*Rbp4*, and *CRABP1*/*Crabp1*, whose expression correlated with the increase in retinol metabolism in wound fibroblasts, as well as on the gene encoding RA receptor β (*RARB*/*Rarb*), a main component and direct target of RAR signaling (30, 31). To separate potential effects of peptide growth factors from retinol and other lipophilic components in the serum, we cultured human primary fibroblasts in medium with either nontreated FBS or FBS that had been preincubated with dextran-coated, activated charcoal (referred to as charcoal-stripped). Activated charcoal adsorbs lipophilic molecules, including retinoids. Although nontreated FBS induced a strong expression of retinol metabolism–associated genes after 24 hours, the effect on *RARB* and *STRA6* was significantly lower with charcoal-stripped FBS (Figure 2A). The reduction was similar to the effect seen with the pan-RAR inhibitor AGN194310 and with the pan-RAR inverse agonist AGN193109, which counteracts the activated and baseline expression of retinol metabolism genes in a dose-dependent manner (Figure 2A and Supplemental Figure 2A) (32). The reduction by charcoal-stripped FBS was almost fully rescued by retinol supplementation of the medium (Figure 2A), indicating that the effect of FBS on the expression of retinol metabolism–associated genes is largely mediated by retinol. Retinol treatment of primary mouse fibroblasts or primary human skin fibroblasts from 2 donors indeed promoted the expression of multiple retinol metabolism genes (Figure 2, B and C). The most responsive gene was *RARB*, the only RAR gene that was directly activated by retinoids (Supplemental Figure 2B). With the exception of *RDH10*, the regulation by retinoids in vitro was similar to the regulation seen in wound fibroblasts. TNF-α, which is highly expressed in early murine skin wounds (33), had only a minor or no effect on the expression of the different retinol metabolism genes (Supplemental Figure 2C). This was also the case for menadione (Supplemental Figure 2D), which induces the production of ROS. In skin wounds, ROS are mainly produced by immune cells during their oxidative burst.

Taken together, these results suggest that the increased expression of retinol metabolism genes in wound fibroblasts results at least in part from their exposure to serum. They also identify retinol itself as a major serum component that mediates this effect.

### Loss of Stra6 has no major effect on wound healing in mice.

**Figure 3:**
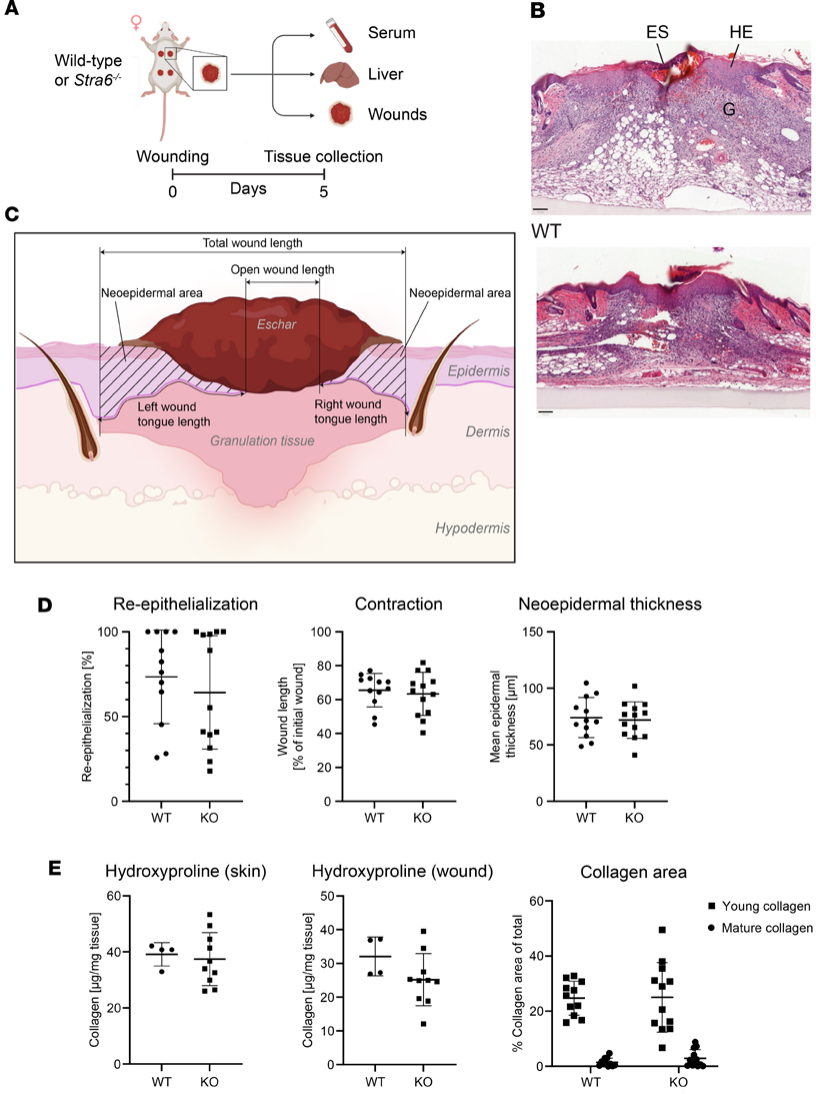
Stra6 is dispensable for wound healing in mice. (**A**) Schematic outline of the experimental setup. Created in BioRender. Werner, S. (2025) https://BioRender.com/fxc8gmk (**B**) Representative images of H&E-stained 5-day wound sections from wild-type (WT) and *Stra6*^–/–^ mice. G, granulation tissue; HE, hyperproliferative wound epidermis; Es, eschar. (**C**) Illustration of wound histomorphometry measurements. Created in BioRender. Werner, S. (2025) https://BioRender.com/714lbmt (**D**) Histomorphometric parameters based on H&E-stained wound sections. *N* = 11–13 mice per genotype (1 wound per mouse). (**E**) Collagen analysis by quantification of hydroxyproline in acid hydrolyzed skin and wound tissues and by quantification of Herovici- (graph “Collagen area”) stained wound sections. *N* = 4–13 mice per genotype (1 wound per mouse). Graphs show mean ± SD. Scale bars: 100 μm.

In tissues that require high concentrations of retinol, such as the retina, STRA6 facilitates the transport of retinol across the plasma membrane (35). The increased expression of Stra6 in wound fibroblasts suggested that retinol uptake via diffusion may not be sufficient and that fibroblasts may require additional retinol uptake via Stra6 during wound healing. To test this hypothesis, we subjected mice with a global knockout of *Stra6* (*Stra6*-KO mice) to full-thickness excisional wounding (Figure 3, A and B). These mice have a vision defect but do not exhibit additional abnormalities under nonchallenged conditions (Supplemental Figure 3A and ref. 35). Histomorphometric analysis (Figure 3C) of H&E-stained sections from the middle of the wounds did not reveal significant alterations in reepithelialization, wound contraction, or neo-epidermal thickness (Figure 3D). Analysis of the hydroxyproline content of wound lysates showed a mild, but nonsignificant, reduction of the collagen levels in the knockout mice. Consistently, deposition of collagen in the granulation tissue and maturation of young to mature collagen, as examined by Herovici staining of wound sections, was unaltered (Figure 3E). Flow cytometry analysis of Pdgfrα^+^ fibroblasts further showed that the number of live wound fibroblasts was not affected, and primary skin fibroblasts isolated from *Stra6*-KO mice had a similar proliferation rate as fibroblasts from wild-type mice within 3 days of culture (Supplemental Figure 3, B and C). RT-qPCR analysis of RNA from whole wounds for *Inhba*, which encodes activin A, an important regulator of wound healing and matrix production (16); *Col1a1*, encoding the major skin collagen; and *Socs3*, one of the main targets of the Stra6/Jak2/Stat5 pathway that is activated by Stra6 (36), showed increased expression of all 3 genes in wounded versus normal skin but no differences between genotypes (Supplemental Figure 3D). Finally, the upregulation of retinol metabolism–associated genes in wound fibroblasts was not affected by the knockout, except for *Stra6* itself (Supplemental Figure 3D). Together, these results reveal that loss of Stra6 does not result in major wound healing abnormalities in mice.

### Mild vitamin A deficiency delays wound healing.

**Figure 4:**
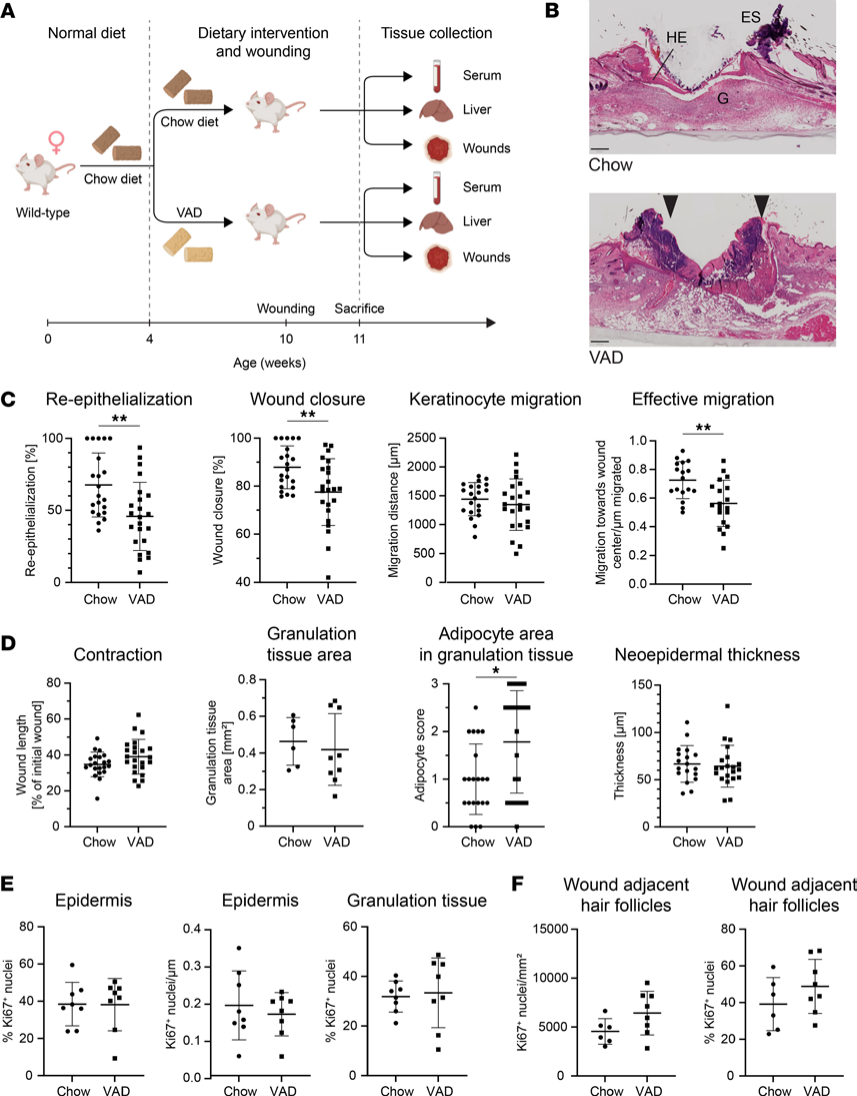
Vitamin A deficiency delays wound healing in mice. (**A**) Schematic outline of the experimental setup. VAD, vitamin A–deficient diet. Created in BioRender. Werner, S. (2025) https://BioRender.com/1psvb1s (**B**) Representative images of H&E-stained sections from 5-day wounds of chow- and VAD-fed mice. G, granulation tissue; HE, hyperproliferative wound epidermis; Es, eschar. Black arrowheads mark wound tongues; chow wound is closed. (**C** and **D**) Histomorphometric parameters based on H&E-stained wound sections of mice fed with VAD or chow diet. *N* = 21–23 mice per treatment group (1 wound per mouse) from 3 independent experiments. Effective migration quantifies the directional keratinocyte migration that contributes to wound closure. (**E**) Percentage of Ki67-positive nuclei and Ki67-positive nuclei/mm^2^ tissue in the epidermis and granulation tissue of wounds from mice fed with VAD or chow diet. *N* = 8 mice per treatment group (1 wound per mouse). (**F**) Percentage of Ki67-positive nuclei and Ki67-positive nuclei per mm^2^ tissue in hair follicles at the wound edge of mice fed with VAD or chow diet. *N* = 8 mice per treatment group (1 wound per mouse). Graphs show mean ± SD. *P < 0.05, **P < 0.01 (Mann-Whitney test). Scale bars: 250 μm.

To further reduce the availability of retinol for fibroblasts and other cells in the wound, we subjected wild-type mice to a vitamin A–deficient diet (VAD) for 6 weeks prior to wounding (Figure 4A) (37). The short diet had no adverse effect and did not lead to significant changes in body weight (Supplemental Figure 4, A and B). The levels of liver retinyl palmitate, one of the main storage forms of retinol, were mildly reduced, likely reflecting the onset of retinol deficiency. However, the difference was not statistically significant because of the high variability in the control mice (Supplemental Figure 4C). As expected from published studies (29, 38), serum retinol levels were not yet reduced at this stage (Supplemental Figure 4D).

Histological and histomorphometric analysis of wound sections showed a significant reduction in reepithelialization and wound closure in VAD- versus chow-fed animals (Figure 4, B and C), while the length of the epithelial tongue, which reflects the extent of migration, was not significantly changed at this time point (Figure 4C). However, the directional migration toward the middle of the wound was significantly impaired in VAD-fed mice (“effective migration”; Figure 4C), providing an explanation for the delayed wound closure. This may point toward impaired migration on the matrix of the newly formed granulation tissue. As contraction strongly contributes to wound closure in mice (39), the normal wound contraction in VAD-treated mice (Figure 4D) allowed an efficient healing process in spite of the impaired reepithelialization. The granulation tissue area in 5-day wounds was similar in mice of both treatment groups (Figure 4D), though the VAD-fed mice showed a significant increase in adipose tissue in the wounds as revealed by semiquantitative blinded wound scoring by investigators (Figure 4D). Epidermal cell proliferation, which is reflected by the neo-epidermal thickness and the number of Ki67-positive epidermal cells (Figure 4, D and E), was not affected by VAD treatment. Furthermore, there were no differences in the rate of proliferation of cells in the granulation tissue and in the hair follicles at the wound edge (Figure 4, E and F), the collagen-positive wound area, the ratio between Picrosirius red–stained collagens and fast green FCF–stained noncollagenous peptides, the hydroxyproline content of the wounds, and the extent of neovascularization (shown by staining with the pan–endothelial cell marker plasmalemma vesicle associated protein, MECA-32) (Supplemental Figure 4, E–H). Despite the significant increase in adipocyte area in the granulation tissue, no significant difference in the content of 16 different lipids were detected (Supplemental Figure 5A).

Taking together, these results show that mild vitamin A deficiency induces defects in wound reepithelialization, which are at least in part mediated by misdirected migration of keratinocytes.

### Wounding alters the abundance of retinol metabolism proteins in the skin.

**Figure 5:**
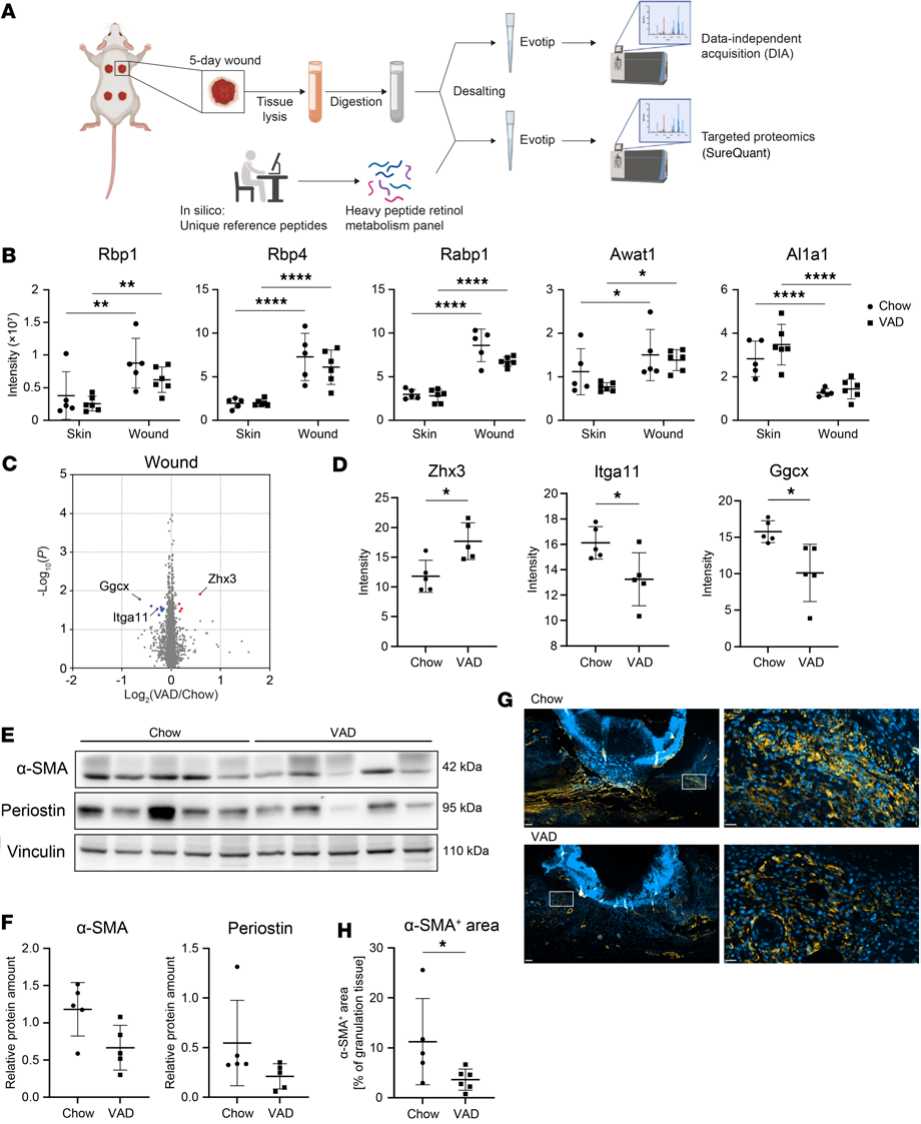
Wounding alters the abundance of retinol metabolism proteins in the skin, and VAD alters the wound proteome. (**A**) Outline of the experimental setup. Created in BioRender. Werner, S. (2025) https://BioRender.com/z88rn2k (**B**) Quantification of significantly differentially abundant retinol metabolism proteins in skin and wound samples of chow-fed or VAD-treated mice based on targeted proteomics analysis (SureQuant). *N* = 5-6 mice. Awat1, acyl-CoA wax alcohol acyltransferase 1. (**C**) Volcano plot showing differentially abundant proteins in lysates from 5-day wounds of chow-fed or VAD-treated mice based on untargeted DIA proteomics analysis. Colored points indicate significantly differentially abundant proteins (*P* < 0.05, |logFC| > 0.15). *N* = 5 mice. (**D**) Quantification of top hits based on DIA analysis in **C**. *N* = 5 mice. (**E**) Western blot for α-SMA, periostin, and vinculin (loading control) using total wound lysates of chow-fed or VAD-treated mice. (**F**) Quantification of the band intensities of **E** and representation as ratio to the respective vinculin loading control. *N* = 5 mice per treatment group. (**G**) Representative images of immunofluorescence stainings for α-SMA (Cy3, orange) of 5-day wound sections of chow-fed and VAD-treated mice from an independent experiment. Hoechst was used to stain nuclei (blue). The area indicated with a white rectangle is shown at higher magnification at the right-hand side. Scale bars: 200 μm (overview) or 20 μm (detail). (**H**) Percentage of the α-SMA^+^ area in the granulation tissue of 5-day wound sections (**G**), determined by quantification of the Cy3-positive area and subtracting the background from the secondary antibody control. *N* = 5–6 mice per treatment group. Graphs show mean ± SD. *P < 0.05, **P < 0.01, ****P < 0.0001. Two-way ANOVA, Šídák’s multiple comparisons test (**B**), or Mann-Whitney test (**D**, **F**, and **H**).

Although obvious alterations in the granulation tissue were not detected, more subtle changes cannot be excluded by these histological data. Therefore, we determined the effect of VAD treatment on the proteome of normal and wounded skin. We first performed targeted proteomics using whole skin and 5-day wound lysates to determine if the increased expression of retinol metabolism genes in wounds translates into alterations at the protein level (Figure 5A). We designed 65 peptides corresponding to 22 proteins associated with retinol metabolism. Thirty-six of these peptides were detected in the skin/wound analysis, of which 34 mapped to a total of 14 proteins. Among the proteins for which 2 peptides were detected, 5 showed a significantly differential abundance in wound versus normal skin tissue (Figure 5B). Consistent with the RNA-Seq data from isolated fibroblasts, Rbp1, Rbp4, and Rabp1 (encoded by the *Crabp1* gene) were more abundant in wound tissue. The abundance of Aldh1a1, one of the rate-limiting proteins in the oxidation of retinaldehyde to RA, was significantly lower in wounds, again following the expression changes observed by RNA-Seq. Awat1, which is involved in the storage of retinyl esters, was significantly more abundant in wounds, while its mRNA was downregulated (Figure 5B). This finding indicates regulation at the protein level or a disparate regulation in fibroblasts compared with other wound cells that were also analyzed in the proteomics experiment. VAD did not significantly alter the abundance of the proteins that we identified by targeted proteomics. Together, these results confirm the RNA-Seq and metabolomics data but do not point to a regulation of retinol metabolism proteins by VAD.

### Vitamin A deficiency alters the granulation tissue proteome.

To determine if VAD affects the wound proteome, we performed quantitative, untargeted data-independent acquisition (DIA) proteomics. A total of 44,197 peptides were identified, which related to 4,451 identified proteins, including some with a significantly differential abundance in wounds of VAD- versus chow-fed controls (*P* ≤ 0.05, |logFC| ≥ 0.15) (Figure 5C). The top hits were Zhx3, a transcriptional repressor with a documented function in the regulation of senescence (40); Itga11, a protein important for adhesion of cells, including myofibroblasts, to the ECM and for wound strength (41); and Ggcx, which catalyzes posttranslational modifications of vitamin K–dependent proteins and is involved in the regulation of glucose metabolism in osteoblasts (42) (Figure 5D). Consistently, Western blot analysis revealed a mild reduction in the levels of the myofibroblast marker α–smooth muscle actin (α-SMA) and of periostin, a matricellular protein that is highly expressed in wounds and fibrotic scars and a potential target for γ-carboxylation by Ggcx (Figure 5, E and F) (43). Immunostaining of wound sections from an independent wounding experiment confirmed that the α-SMA^+^ area in the granulation tissue was significantly reduced in VAD-treated mice (Figure 5, G and H). Together, these findings support the hypothesis that VAD alters the composition of the wound granulation tissue.

**Figure 6:**
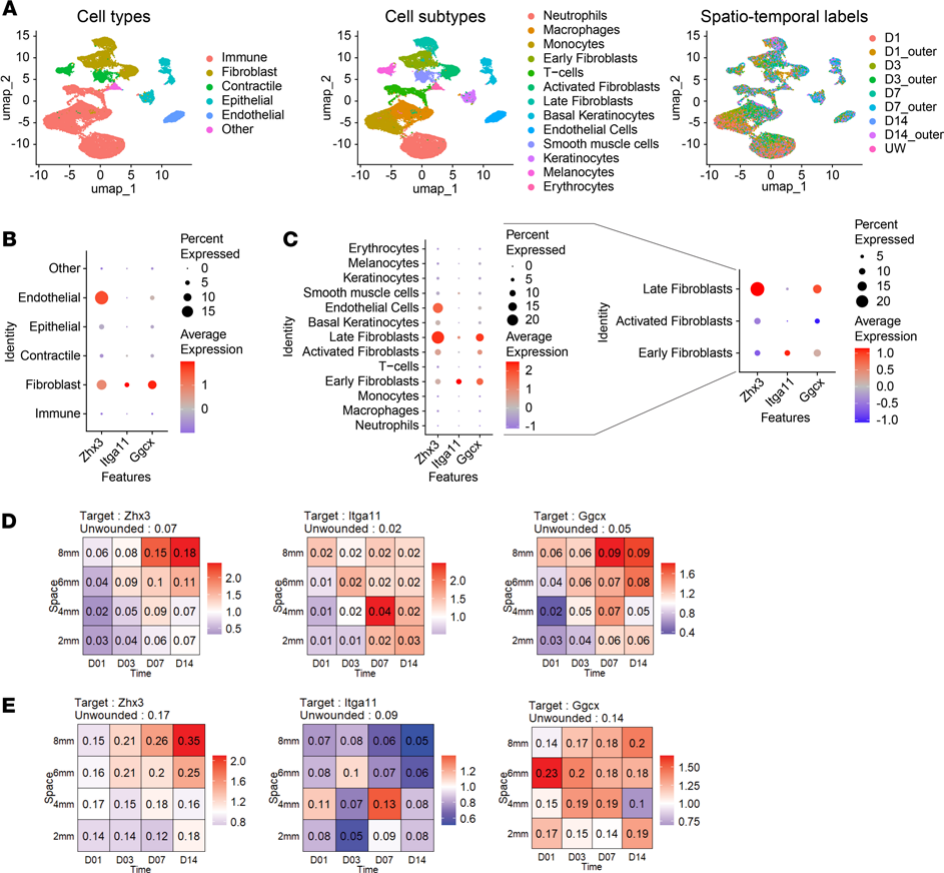
Proteomics hit proteins are mainly expressed in wound fibroblasts. (**A**) Uniform manifold approximation and projection (UMAP) visualization of wound cells and their annotations to cell types (left panel), subtypes (middle panel), and spatiotemporal labels (right panel) based on a mouse wound dataset (26). (**B**) Breakdown of expression of *Zhx3*, *Itga11*, and *Ggcx* in all annotated cell types. Dot size correlates with the percentage of cells that express the gene of interest, while color represents their average expression levels. (**C**) Breakdown of expression of *Zhx3*, *Itga11*, and *Ggcx* in all annotated cell subtypes (left) and specifically in the annotated fibroblast subtypes (right). Dot size correlates with the percentage of cells that express the gene of interest, while color represents their average expression levels. (**D** and **E**) Spatiotemporal expression of *Zhx3*, *Ggcx*, and *Itga11* in all cells (**D**) and in the fibroblast subcluster (**E**). Space on the y axis refers to increasingly large rings around the wound center; wounds were sampled on day 1, 3, 7, and 14 as depicted on the x axis. The number in the square indicates the percentage of the cell subpopulation among all cells. Background color indicates relative change compared with the unwounded state (red: increase, blue: decrease, white: no change).

We next analyzed published scRNA-Seq data, which had been obtained at different time points post wounding and with tissue of different distance from the middle of the wound (26), to determine if the identified hits are expressed by wound fibroblasts. Figure 6A depicts a uniform manifold approximation and projection (UMAP) visualization of the different cell types, subtypes, and spatiotemporal labels defined in the scRNA-Seq reanalysis. Zhx3, Itga11, and Ggcx transcripts were mainly detected in fibroblasts (Figure 6B). While *Itga11* was exclusively expressed in cells with an early fibroblast expression profile, *Ggcx* was expressed in early, late, and activated fibroblasts (Figure 6C). *Zhx3* mRNA was highly enriched in cells with a late fibroblast profile but also showed moderate expression levels in endothelial cells. We re-created spatiotemporal expression profiles of the 3 proteomics hits in all cells and in fibroblasts. Expression of *Zhx3* increased on day 7 in the outer areas of the wound and was strongly expressed until day 14. *Itga11* expression peaked near the wound center on day 7 after wounding. *Ggcx* showed the highest expression in the outer areas of the wound (Figure 6, D and E).

Finally, we tested whether the 3 hit proteins are direct targets of RA signaling. Treatment of primary human fibroblasts or immortalized mouse fibroblasts with retinol, RA, or the pan-RAR inverse agonist AGN193109 in the presence or absence of serum did not affect the expression of *GGCX* and *ZHX3*, while *RARB* and *CRABP1* were strongly regulated. *ITGA11* expression was even mildly reduced by RAR signaling in primary human fibroblasts (Supplemental Figure 6, A–C). The 3 hit genes were also not consistently regulated by retinols in cultured mouse fibroblasts (Supplemental Figure 6, D and E). Together, these findings indicate that the altered abundance of GGCX, ITGA11, and ZHX3 in wounds of VAD-treated mice results from alterations in the fibroblast phenotype and not from a direct effect of retinoids on their expression. The delayed reepithelialization of wounds in VAD-treated mice is a likely consequence of the alterations in fibroblast-specific proteins and concomitant changes in the granulation tissue composition.

## Discussion

In this study, we identified retinol metabolism as a key pathway in wound healing. Notably, we observed enrichment of retinol and of retinol and RA binding proteins. The genes for 2 of them, *Crabp1* and *Fabp5*, had been identified as markers for regenerative fibroblasts in scRNA-Seq analyses of mouse wounds, suggesting that the increase in retinol metabolism is important for repair (9, 44, 45). Analysis of published RNA-Seq data from mouse wounds at different time points confirmed the activation of retinol metabolism–associated genes in the proliferative phase of wound healing, in particular in fibroblasts. Consistent with an important role of retinol metabolism in wound healing, the efficiently regenerating MRL/MpJ mouse line exhibits increased expression of genes involved in retinol metabolism (46). Vice versa, serum retinol levels were reduced in patients with burn wounds or chronic leg ulcers (47, 48). Therefore, retinoids have been tested for their efficacy to stimulate wound healing (49). In most studies, topical retinoid treatment promoted repair in healing-impaired male or female mice or rats (50–52). In wounds of healthy pigs, topical tretinoin (all-*trans* RA) treatment retarded while tretinoin pretreatment promoted healing (53). In a human study, short-contact treatment of chronic leg ulcers with tretinoin promoted granulation tissue formation in male and female patients (54). Different effects of retinol and its derivatives on myofibroblast differentiation were also observed. While VAD reduced the number of myofibroblasts in mouse wounds in vivo (this study), retinoids inhibited and RAR antagonists promoted contraction of mouse fascia explant cultures (55). In a bleomycin-induced skin fibrosis model, inhibition of RA catabolism and the resulting increase in RA signaling inhibited fibrosis and shifted fibroblast populations towards a regenerative state (56). Therefore, different fibroblast subtypes may respond differently to retinoids, which could be further influenced by the in vivo environment. Together, these results indicate that retinol metabolism and RA signaling in wounds must be spatially and temporally tightly regulated and that they depend on the underlying pathology.

In contrast to these insightful treatment studies, the consequences of retinol deficiency for wound healing are still largely unknown. Here we show that the knockout of *Stra6* has no major effect on wound healing in mice, suggesting that retinol uptake by diffusion is largely sufficient. While *Stra6*-KO mice only show a disruption of the visual cycle (35), truncating STRA6 mutations in humans are associated with severe organ malformations and growth retardations (57, 58). These findings point to a different importance of *Stra6*/*STRA6* in mouse versus human tissue and suggest that STRA6 deficiency may have more severe consequences for wound healing in humans.

To further decrease the availability of retinol for wound fibroblasts, we subjected wild-type mice to a 6-week VAD. We refrained from prolonging the diet and thereby inducing a pathological vitamin A deficiency, as the resulting systemic abnormalities and malady would lead to unspecific wound healing defects. Serum retinol levels only decline after the liver stores of retinyl esters are depleted, but consequences of vitamin A deficiency were already observed before significant changes in total serum retinol were detected (29, 38). This may be explained by differences in the uptake of different retinol complexes. While retinol is almost exclusively bound to Rbp4 under fasting conditions, it is found in the form of retinyl esters in chylomicrons postprandially. Most of these chylomicrons are taken up by the liver, where retinol is stored for later distribution through Rbp4. However, some extrahepatic tissues, including the skin, may take up a substantial portion of their retinol from chylomicrons (59). The lack of this supply in VAD-treated mice provides a possible explanation for their wound healing abnormalities.

The delay in wound closure in VAD-treated mice was mainly a consequence of impaired directional migration of keratinocytes, which may result from alterations in the contact with the underlying matrix. Consistent with this assumption and despite the systemic VAD treatment, proteomics analysis of wound tissue identified fibroblast-enriched proteins as the primary hits. This result points to alterations in fibroblasts and differences in the composition of the granulation tissue as major drivers of the healing defect in VAD-treated mice. In a recent study, VAD treatment of adult mice affected hair follicle stem cell plasticity, which was associated with delayed reformation of the epidermal barrier after wounding (60). Although wound closure, reepithelialization, and granulation tissue formation were not analyzed in that study, the data suggest an additional cell-autonomous effect of retinol deficiency in keratinocytes on wound reepithelialization.

Among the significantly less abundant proteins in wounds of VAD-treated mice was Itga11, a collagen receptor involved in migration and ECM reorganization (61, 62). Upregulation of *ITGA11* expression increased migration of cancer-associated fibroblasts, while reduced expression of this gene in dermal fibroblasts impaired their migratory capacity (63, 64). Itga11 expression was induced by mechanosignaling in the skin and is important for myofibroblast differentiation in skin and liver (65, 66). Most importantly, wounds in *Itga11*-knockout mice had less granulation tissue, lacked myofibroblasts, and showed reduced contractility and tensile strength (41). These findings, together with the reduced levels of Itga11 and α-SMA in wounds of VAD-treated mice, support the notion that myofibroblast differentiation is impaired in wounds of VAD-treated mice. The resulting alterations in the granulation tissue provide a likely explanation for the impaired directional migration of keratinocytes.

Ggcx, a vitamin K-dependent γ-glutamyl carboxylase, was also less abundant in wounds of VAD-treated mice. Gamma-carboxylation is an important protein modification that activates specific proteins (67). Its reduction might affect periostin, which requires γ-carboxylation in some, but not all tissues (68, 69). In skin wounds, periostin facilitates cell-matrix interactions by binding to α_V_β_3_ and α_V_β_5_ integrins and modulates myofibroblast differentiation (43, 70).

In conclusion, we applied multiple omics technologies to identify important pathways in wound fibroblasts. We also established a targeted proteomics strategy that allows the identification of multiple components of the retinol metabolism pathway in any tissue. Therefore, our work provides important datasets and tools for the study of wound healing and retinol metabolism. Most importantly, we identified retinol metabolism as a key pathway in wound healing, which opens avenues for its precise manipulation for the treatment for impaired healing.

## Methods

### Sex as a biological variable

The wound healing studies were only performed with female mice, because male mice often have bite wounds and scars that may affect the healing process and because previous studies showed similar effects of retinoids on wound healing in male and female rodents and humans. While the overall mechanisms of wound healing are comparable between sexes, differences in RA metabolism between female and male mice during wound healing cannot be excluded.

### Mice

RNA-Seq and metabolomics experiments were performed with skin and wound tissue from wild-type mice in CD1 genetic background. *Stra6*-KO mice (35) and wild-type C57BL/6 mice of the same breedings were used for wound healing studies. For VAD experiments, wild-type C57BL/6 mice were either from in-house breeding or purchased from Elevage Janvier. Mice were housed under specific pathogen–free conditions in a 12-hour dark/12-hour light cycle. They received water and food ad libitum.

### Mouse genotyping

Genomic DNA was isolated from biopsies obtained from ear clipping (16). *Stra6* alleles were identified as described (35). Primers are detailed in Supplemental Table 2.

### VAD

VAD was started at the age of 4 weeks until the wound healing experiment was performed at the age of 10 weeks. The food retinol content was guaranteed to be lower than 0.22 IU/g according to the manufacturer (Hespra Bio and Kliba Nafag). Control animals received a normal chow diet with the same ingredients but containing 23.1 IU/g retinol.

### Wound healing experiments

Wound healing experiments were performed as previously described (16). Wounds were collected using a 5 mm punch biopsy, and the subcutaneous fat was removed. For stainings, wounds were bisected to obtain sections from the middle of the wound.

### FACS isolation of skin and wound fibroblasts

FACS isolation of fibroblasts, including the gating strategy, was previously described (16). Between 80,000 and 100,000 cells were sorted. For metabolomics, they were washed with ammonium carbonate wash solution (75 mM ammonium carbonate in H_2_O, pH 7.4, freshly prepared) and centrifuged at 1,000*g* for 10 minutes. The supernatant was removed, and the cells were snap-frozen in liquid nitrogen and stored at –80°C.

### RNA isolation and RT-qPCR from tissues

Total RNA was isolated from shock-frozen tissue pieces using TRIzol. Reverse transcription was performed with 500–1,000 μg of RNA using the iScript cDNA synthesis kit (Bio-Rad). RT-qPCR (16) was performed using primers detailed in Supplemental Tables 4 and 5.

### Western blot analysis

RIPA buffer (300 μL) was added to snap-frozen tissue samples. The tissue was disrupted with a TissueLyser II with shaking at 30 Hz for 5 minutes. Protein concentration was determined using the BCA assay (Thermo Fisher Scientific). Protein concentrations were adjusted with RIPA buffer, and samples were denatured by addition of Laemmli buffer and incubation at 95°C for 5 minutes. Proteins were separated by polyacrylamide gel electrophoresis and transferred to nitrocellulose membranes. Unspecific binding sites were blocked by incubation in 5% skim milk in TBS with Tween 20 for 1 hour, and the membranes were incubated with the primary antibody overnight (o/n) at 4°C. After washing, HRP-coupled secondary antibodies were added for 1 hour at room temperature (RT). Antibodies used are shown in Supplemental Table 3.

### Metabolomics

FACS-sorted cells were stored at –80°C and extracted with 50 μL cold (–20°C) 40:40:20 acetonitrile/methanol/water. After 10 minutes of incubation at –20°C with sporadic vortexing, cells were pelleted by centrifugation at 4°C and full speed in a table-top centrifuge. The clear supernatant (20–30 μL) was transferred to a plate for injection into the mass spectrometer. Untargeted metabolomics by flow injection mass spectrometry (MS) was performed as described (71). The samples were injected at an isocratic flow rate of 150 μL/min of isopropanol/H_2_O (6:4, v/v) containing ammonium fluoride (1 mM) and the reference compounds hexakis (2,2,3,3-tetrafluoropropoxy)phosphazene and homotaurine (3-amino-1-propane sulfonic acid). Electrospray ionization was used with the following source parameters: gas temperature 225°C, drying gas 11 L/min, nebulizer 20 psi, sheath gas temperature 350°C, sheath gas flow 10 L/min, Vcap 3,500 V, and nozzle voltage 2,000 V. The fragmentor was set at 350 V, and the Oct1RF Vpp was set at 650 V. The mass spectrometer was operated in full scan mode scanning the mass range (50–1,000 *m/z*) at 1.4 spectra per second. An online mass correction was performed using the reference masses 138.0230374 and 940.0003763.

Raw MS data were converted to an open-source format (mz5) and processed using an in-house data processing pipeline (FiaMiner) in Matlab (MATHWORKS). The *m/z* axis for the whole dataset was recalibrated using expected masses. Afterward, annotation was performed based on *m/z*, with mass accuracy of 0.001 Da matching to the human metabolome database. Differential analysis statistics was performed in Matlab for pairwise comparisons followed by correction of multiple comparisons.

### Determination of retinoid content by targeted MS

#### Liquid-liquid extraction.

All procedures were performed in the dark. Samples were thawed on ice and briefly vortexed. Protein content of the 1:100 diluted samples was measured using a Lunatic spectrophotometer (Unchained Labs). A total of 70 μL of each sample was transferred to a glass tube, 7 μL of 1.1 M HCl and 578 μL of methanol (MeOH) were added and vortexed, 1,925 μL of methyl *tert*-butyl ether were added, and the mixture was vortexed again and incubated for 15 minutes at 25°C in a sonication bath. Then 481 μL of Milli-Q water (MilliporeSigma) were added, vortexed, and centrifuged for 5 minutes at 6,250*g* and 4°C. The upper organic phase was collected and dried under a nitrogen stream at 30°C. The aqueous phase and pellets were stored at –80°C. The evaporation residues of the organic phase were reconstituted in 105 μL 75% MeOH/0.1% formic acid, vortexed, and subjected to shaking at 1,400 rpm for 15 minutes at 15°C, before being vortexed again. The solution was centrifuged at 18,500*g* for 10 minutes at 4°C, and the supernatant was analyzed by liquid chromatography-tandem mass spectrometry on a triple-quadrupole mass spectrometer using selected reaction monitoring (for details see Supplemental Tables 6 and 7).

### Lipidomics

For quantitative lipidomics, internal standards were added prior to lipid extraction. Tissue samples of 2 mg wet weight were subjected to lipid extraction (72). Lipid analysis was performed by direct flow injection analysis (FIA) using a triple-quadrupole mass spectrometer (FIA-MS/MS, Quattro Ultima) and a high-resolution hybrid quadrupole-Orbitrap mass spectrometer (FIA-FTMS, Thermo Fisher Scientific). FIA-MS/MS was performed in positive ion mode using the analytical setup and strategy described previously (73). A fragment ion of *m/z* 184 was used for lysophosphatidylcholines (74). The following neutral losses were applied: phosphatidylethanolamine (PE) and lysophosphatidylethanolamine 141, phosphatidylserine 185, phosphatidylglycerol (PG) 189, and phosphatidylinositol 277 (75). Sphingosine-based ceramides and hexosylceramides were analyzed using a fragment ion of *m/z* 264 (76). PE-based plasmalogens were analyzed according to the principles described by Zemski Berry (77). Cardiolipin was monitored by diglycerol fragment ions (78). Glycerophospholipid species annotation was based on the assumption of even-numbered carbon chains only.

FIA-FTMS was performed as described (79). Triglycerides, diglycerides, and cholesterol esters (CE) were recorded in positive-ion mode *m/z* 500–1,000 as [M+NH_4_]^+^ at a target resolution of 140,000 (at 200 *m/z*). CE species were corrected for their species-specific response (80). Phosphatidylcholines (PC), PC ether, and sphingomyelins were analyzed in negative ion mode *m/z* 520–960 as [M+HCOO]^–^ at the same resolution setting. Analysis of free cholesterol (FC) was performed by multiplexed acquisition of the [M+NH_4_]^+^ of FC and the deuterated internal standard (FC[D7]) (80). Free fatty acids were analyzed in negative ion mode *m/z* 150–450 as [M-H]^–^ dissolved in MeOH/chloroform 5:1 (v/v) containing 0.005% dimethylamine.

### Bioinformatic analysis of RNA-Seq and metabolomics data

#### Overrepresentation analysis.

Overrepresentation analysis was performed using ClusterProfiler in RStudio 2023.06.0+421 (R version 4.3.2) (81). Differentially expressed genes were defined by a |log_2_(fold change)| over 0.5 and a *P* value below 0.05.

#### Integrated pathway analysis.

A joint pathway analysis was performed using MetaboAnalyst 6.0 (82). Significantly differentially expressed genes (*P* ≤ 0.05) and significantly differentially abundant metabolites (*P* ≤ 0.05) found in both experiments were used for the analysis.

### Bioinformatic analysis of scRNA-Seq data sets

The scRNA-Seq data sets from mouse skin wounds were downloaded from Gene Expression Omnibus (GEO) (GSE204777) (26) and reanalyzed using the Seurat R package (version 4.2.0). The data from the 5 batches were first analyzed separately for quality control by eliminating low-quality cells with low RNA features, as well as those with relatively high mitochondrial and ribosomal RNA content via the subset function, followed by *SCtransform* using the *vars.to.regress* modifier to additionally regress out the mitochondrial and ribosomal RNA features (83). Time point– and wound location–specific barcodes were determined for each cell using the HTODemux hash tag oligo demultiplexing function. The data sets were merged and integrated using the *merge*, *SelectIntegrationFeatures, PrepSCTIntegration, FindIntegrationAnchors* and *IntegrateData* functions, according to the published data analysis and integration protocol (84). The integrated data sets were analyzed via the *RunPCA* function, followed by *ElbowPlot* to visualize the standard deviation of the principal components and to select the most significant dimensions for further analyses. The *RunUMAP* and *FindNeighbors* functions were run using standard settings and the *dims=1:30* modifier, followed by *FindClusters* with *resolution=0.1* to cluster the data set on major cell types. Marker genes of skin cells were obtained from CellMarker 2.0 (85) and mapped onto the data set by *DotPlot* and *FeaturePlot*. Immune cells, fibroblasts, contractile cells, epithelial cells, endothelial cells, and others were identified and annotated. Using additional cell markers, the following subtypes of the major cell types were identified and annotated: neutrophils, macrophages, monocytes, early fibroblasts, T cells, activated fibroblasts, late fibroblasts, basal keratinocytes, endothelial cells, smooth muscle cells, keratinocytes, melanocytes, and erythrocytes. Wound fibroblasts were analyzed by first subsetting the fibroblast clusters via the *Subset* function, followed by the downstream steps outlined above. Marker genes for fibroblast subtypes were determined by the *FindAllMarkers* function. Space/time tile plots for individual genes were generated as described (26).

### IHC

FFPE samples were dewaxed, rehydrated, and washed with PBS. Antigen retrieval was performed with 0.1 M sodium citrate buffer (pH 6) at 95°C in a water bath and incubation for 1 hour. After 30 minutes of cooling at RT and washing, slides were incubated in blocking buffer (12% BSA in PBS with Tween 20) for 1 hour at RT. The primary antibody in blocking buffer was incubated at 4°C o/n. After washing, the biotinylated secondary antibody was added in blocking buffer for 1 hour at RT. Developing was performed as described by the manufacturer of the VECTASTAIN Elite ABC-HRP Kit (Vector Laboratories). After washing with water, sections were counterstained with hematoxylin for 3 minutes, dehydrated, and mounted with Mowiol. Antibodies used are specified in Supplemental Table 3.

#### Quantification of histological stainings.

Quantification of Herovici staining was performed using a custom script in ImageJ (NIH) (86). Light blue pixels resulting from young (type III–enriched) collagen fibers stained with methyl blue, while dark purple pixels resulting from matured (type I–enriched) collagen fibers stained with acid fuchsin (87). Areas with these colors of interest were manually selected to define color ranges for later pixel-based analysis, and the granulation tissue was manually segmented. Within the segmented area, the chosen color ranges were used to define pixels positive for young or mature collagen, respectively. The same color segmentation setting was applied to all images. The positively stained pixels were plotted as percentage of total pixels within the total granulation tissue area.

Ki67 staining was quantified using QuPath 0.5.0 (88). Ki67^+^ cells in the wound epidermis were quantified using the positive cell detection feature of QuPath and then calculating the percentage of positive cells among all cells and the number of positively stained epidermal cells per micrometer or millimeter of epidermis.

Collagenous and noncollagenous peptides in Picrosirius red/fast green FCF-stained sections were quantified using QuPath 0.5.0. Red (collagenous peptides, Picrosirius red) and green (noncollagenous peptides, fast green FCF) pixels were thresholded in the manually segmented granulation tissue, and positively classified areas were measured. Collagen area ratio was calculated by taking the ratio of the Picrosirius red–positive and the fast green–positive area.

MECA-32 and α-SMA stainings were analyzed using QuPath 0.5.0. For quantification of the α-SMA and MECA-32–positive areas, the granulation tissue was manually segmented. The MECA-32– (Cy3) positive area was determined by thresholding and normalized to the granulation tissue area. To establish a threshold for specific signal detection and minimize background interference in the α-SMA staining, the mean gray value and SD of the nonspecific signal seen in the secondary antibody control were quantified. A cutoff value was defined as the mean gray value plus twice the SD (μ + 2σ). Signals in the stained sections exceeding this threshold were considered specific, while those below were attributed to background and excluded from analysis. The α-SMA–positive area was normalized to the granulation tissue area.

### Proteomics

#### Protein lysate preparation.

Snap-frozen wound and skin samples were transferred into 400 μL 4 M guanidine hydrochloride in 250 mM HEPES, pH 7.8. Metal beads were added, and the tissues were lysed using a TissueLyser II at 30 Hz in 2 cycles of 2 minutes, followed by 1 cycle of 1 minute. Samples were sonicated for 10 cycles of 30 seconds on/30 seconds off at 4°C and centrifuged at 10,000*g* for 10 minutes at 4°C. A volume equivalent to 20 μg of protein was diluted to 40 μL with lysis buffer. For this purpose, 1 μL of 350 mM Tris(2-carboxyethyl)phosphine was added to a final concentration of 10 mM, followed by the addition of 2.8 μL of freshly prepared 500 mM chloroacetamide solution to a final concentration of 40 mM. The mixtures were incubated for 5 minutes at 95°C and then diluted to 2 M guanidine hydrochloride with 45 μL of 250 mM HEPES buffer. Subsequently, 0.5 μL of a 1 μg/μL of Lys-C endoproteinase stock solution was added to achieve an enzyme/protein (w/w) ratio of 1:40, and the samples were digested for 3 hours at 37°C while shaking at 350 rpm. They were further diluted to 0.5 M guanidine hydrochloride by adding 240 μL of 250 mM HEPES buffer, before adding 1 μL of a 1 μg/μL trypsin stock solution to achieve an enzyme/protein lysate (w/w) ratio of 1:20. Samples were incubated o/n at 37°C with shaking at 350 rpm. Trypsin was inactivated by acidifying the samples with approximately 90 μL of 10% trifluoroacetic acid (TFA) to achieve a final concentration of around 1% TFA and a pH of 1–2.

#### EvoTip preparation and sample loading.

We added 20 μL of acetonitrile to each EvoTip column, followed by centrifugation at 700*g* for 1 minute. Tips were soaked in 100% isopropanol for 20 seconds. While soaking, 20 μL of 0.1% formic acid (FA) was added to the top of the tips, and the solution was removed by centrifugation at 700*g* for 1 minute. A sample containing 750 ng of protein was diluted to a final volume of 20 μL with 0.1% FA and added to the tips. The tips were centrifuged at 700*g* for 1 minute, followed by 2 washes with 20 μL 0.1% FA, each step including centrifugation at 700*g* for 1 minute. After the second wash, 250 μL of 0.1% FA was added to the top and centrifuged at 700*g* for 10 seconds. The loaded tips were stored soaked in 0.1% FA until analysis.

#### DIA data acquisition.

The analysis was conducted using an Orbitrap Exploris 480 mass spectrometer (Thermo Fisher Scientific) in combination with an LC Evosep One system. Samples were run using the pre-programmed Whisper100 10SPD method on an Acclaim PepMap RSLC C18 column (1.9 μm, 75 μm × 150 mm, Thermo Fisher Scientific). The mass spectrometer operated in positive ion mode and was coupled with a FAIMS Pro interface, with the compensation voltage set to –45 V. MS scans were recorded at a resolution of 120,000, covering a mass range of 400–1,000 *m/z*, with an AGC target of 300% and maximum injection time set to auto. The DIA isolation windows also spanned 400–1,000 *m/z*. Precursor ions were fragmented using higher-energy collisional dissociation (HCD) with a normalized collision energy of 32%. Fragment ion scans were acquired at a resolution of 60,000, with an automatic gain control (AGC) target of 1,000% and the maximum injection time set to auto.

#### DIA data analysis.

Raw DIA data files were analyzed using Spectronaut 17 (Biognosys) in DirectDIA (Deep) mode, utilizing the mouse UniProt database (taxonomy ID: 10090) with modified Biognosys factory settings. The enzyme specificity was set for database searches to trypsin and Lys-C, allowing up to 1 missed cleavage. Carbamidomethylation of cysteine was selected as a fixed modification, while acetylation of the protein N-terminus and methionine oxidation were considered variable modifications. Quantification was performed at the MS1 level, focusing exclusively on proteotypic peptides. Protein quantification data were extracted and subjected to further analysis using a custom Python script (Python version 3.11.4), which also performed the 2-way ANOVA for statistical assessment.

#### SureQuant analysis.

Three peptides per protein, which identify both human and mouse orthologs of the proteins (Supplemental Table 8), were chosen for assay validation and suitability. Human reference peptides were taken from Proteomics DB (https://www.proteomicsdb.org/proteomicsdb/#human), and mouse reference peptides were identified from https://prospector.ucsf.edu/prospector/cgi-bin/msform.cgi?form=msdigest Whenever possible, 3 peptides present in both reference peptide sets were used. The final selection of peptides was based on a defined set of criteria, including frequent and high-confidence identification in MS experiments. The peptides were required to meet specific conditions: absence of methionine, a length between 8 and 21 amino acids, a C-terminal arginine or lysine, and no missed cleavages, whenever possible. The heavy isotope–labeled arginine or lysine peptides, used for SureQuant analysis, were purchased from JPT Peptide Technologies.

#### SureQuant data acquisition.

SureQuant analysis was performed on an Orbitrap Exploris 480 mass spectrometer (Thermo Fisher Scientific) in combination with an LC Evosep One system. Samples were run using the pre-programmed 88-minute gradient on an Acclaim PepMap RSLC C18 column (1.9 μm, 75 μm × 150 mm, Thermo Fisher Scientific). A total of 750 ng of sample along with 500 fmol of heavy peptides was used for the analysis. The MS1 scan was performed with a resolution of 120,000, an AGC target of 300%, an injection time of 50 ms, and a cycle time of 3 seconds. This was followed by the detection of heavy peptide precursors and their fragments based on a predefined list (selected proteins and respective peptides are detailed in Supplemental Table 8), using a resolution of 7,500, HCD energy of 30, AGC target of 1,000%, and a 10 ms injection time. The product ion trigger required detection of at least 2 product ions to initiate an offset scan, which was carried out at a resolution of 120,000, HCD energy of 30, AGC target of 1,000%, and a 250 ms injection time in profile mode.

#### SureQuant data analysis.

The SureQuant data were analyzed using Skyline version 23.1. Target peptides were imported with the MS1 filter parameters set to centroided precursor mass analyzer, mass accuracy of 10 ppm, and MS/MS filter parameters set to SureQuant acquisition method, with Orbitrap as a product mass analyzer at a resolution of 60,000 at 200 *m/z*. Each precursor mass was configured to monitor at least 3 product ions. All peptides included a fixed modification of carbamidomethylation on cysteine (+57.021 Da) and oxidation on methionine (+15.994 Da). Synthetic peptides also had static modifications with heavy isotopes, lysine (+8.014 Da) and arginine (+10.008 Da). Peptide transition peaks were reviewed and adjusted manually using the retention times of the heavy peptides as references. Peptide fragment areas were normalized to the total ion current chromatogram area. The total area fragment for each precursor was used for quantification. Protein quantification was performed by averaging the values of its detected peptides.

### Statistics

All statistical data are based on biological replicates. Statistical analysis was performed with Prism software, version 10 for Windows (GraphPad Software). Mann-Whitney *U* test was used for the comparison of 2 groups. For comparisons involving more than 2 groups, we used Šídák’s or Tukey’s multiple comparisons test as specified in the figure legends. A *P* value of less than 0.05 was considered significant.

### Study approval

Mouse experiments and maintenance of mice were approved by the local veterinary authorities (Kantonales Veterinäramt Zürich, Zurich, Switzerland).

### Data availability

RNA-Seq data (16) had been deposited at GEO. They are available under the accession number GSE134789.

The proteomics data had been submitted to the ProteomeXchange Consortium via the PRIDE repository and are available under the accession codes PXD061167 (DIA dataset) and PXD061256 (SureQuant dataset) (89, 90).

Raw data of the metabolomics analysis had been uploaded to MassIVE and are available under accession number MSV000097301 (ftp://massive.ucsd.edu/v09/MSV000097301/).

All original data used to create the figures of this manuscript are available in the Supporting Data Values file.

Further information can be found in Supplemental Methods.

## Author contributions

TW designed and performed all cell culture and wound healing experiments, designed the proteomics experiment with EM and UADK, conducted the proteomics experiment and analysis with EM, performed the integration of the bulk RNA-Seq and metabolomics experiments, and wrote the manuscript together with SW. SW led the study from conception to design of experiments, provided troubleshooting, and supported analysis of the data. EM performed the DIA analysis. MSW performed the FACS experiment and reanalyzed published scRNA-Seq data. NBG provided *Stra6*-KO mice and expertise in retinoid biology. MH and GL designed, performed, and analyzed the lipidomics experiments. NZ designed and performed the metabolomics experiment. All authors provided important feedback to the manuscript.

## Funding support

Swiss National Science Foundation (310030-212212 to SW).

## Supplementary Material

Supplemental data

Unedited blot and gel images

Supporting data values

## Version 1 10/16/2025

In-Press Preview

## Version 2 11/24/2025

Electronic publication
