# Supplementary material for: A multiomics analysis identifies retinol metabolism in fibroblasts as a key pathway in wound healing
Source: JCI Insight. 2025 Oct 16;10(22):e194188. doi: 10.1172/jci.insight.194188 (PMC12643503; doi:10.1172/jci.insight.194188)
Supplement: Supplemental data [file jciinsight-10-194188-s177.pdf]

Supplemental Information

Wüstemann et al.

Supplemental Figures

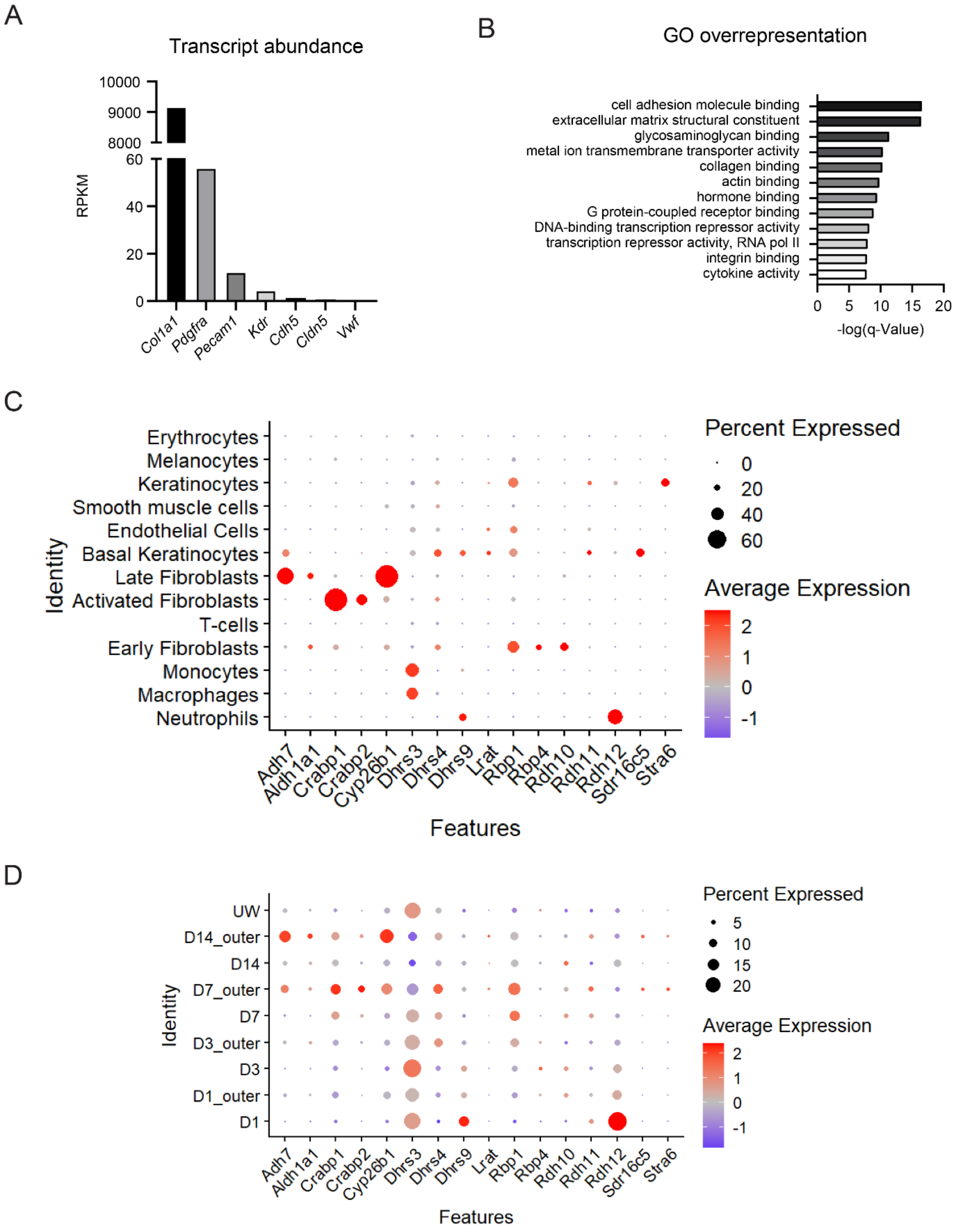

Supplemental Figure 1: Gene ontology (GO) overrepresentation of pathways in wound fibroblasts and temporal analysis of retinol metabolism-associated genes in wound cells.

A. Fibroblast (*Col1a1*, *Pdgfra*) and endothelial cell (*Pecam1*, *Kdr*, *Cdh5*, *Cldn5*, *Vwf*) marker expression in the RNA-Seq data set. RPKM: Reads per kilobase per million mapped reads.

B. Gene ontology (GO) overrepresentation analysis of the RNA-Seq data from Figure 1C.

C. Analysis of the cell-specific expression of retinol metabolism-associated genes in a published scRNA-Seq data set (26).

D. Analysis of the temporal expression of retinol metabolism-associated genes in the same data set. Expression levels are analyzed in unwounded (UW), day 1 (D1), day 3 (D3), day 7 (D7), and day 14 (D14) wound samples. The “outer” suffix refers to the skin adjacent to the wounds.

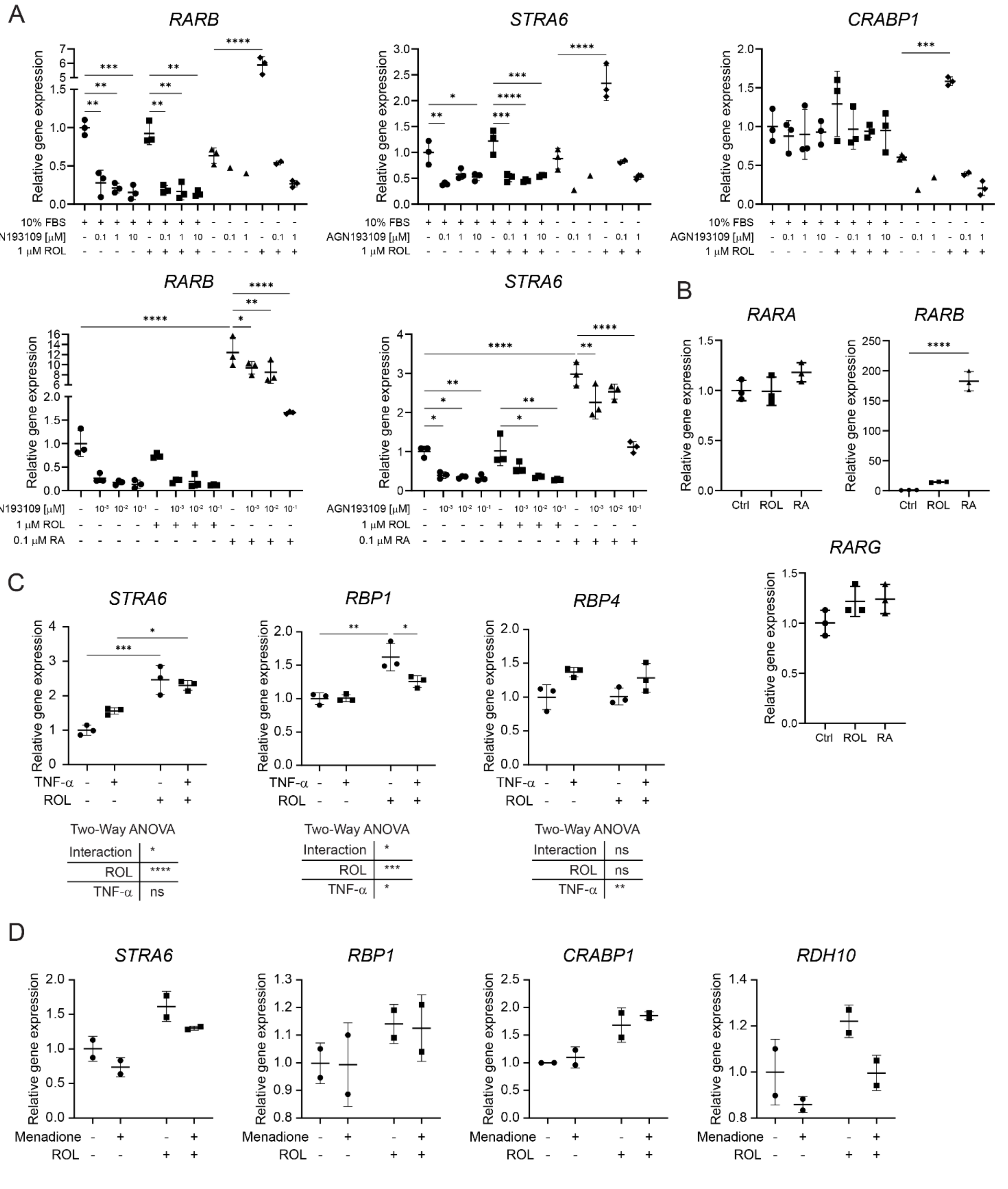

Supplemental Figure 2: Changes in retinol metabolism upon retinoid treatment are RAR dependent.

A. RT-qPCR for *RARB*, *STRA6*, and *CRABP1* relative to *RPL27* using RNA from primary human fibroblasts, treated with 1 μM retinol (ROL) or RA, or different concentrations of

AGN193109 (AGN) for 24 h after 48 h of starvation in serum-free medium. N=1-3 cultures from 1 donor per treatment group.

B. RT-qPCR for *RARA*, *RARB* and *RARG* using RNA from primary human fibroblasts of donor 2, treated with 1 µM ROL or RA for 24 h, after 48 h of starvation in serum-free medium. N=3 cultures from 1 donor per treatment group.

C. RT-qPCR for *STRA6*, *RBP1* and *RBP4* using RNA from primary human fibroblasts, treated with 1 µM ROL or 10 ng/ml TNF-α for 24 h after 48 h starvation in serum-free medium. N=3 cultures from 1 donor per treatment group.

D. RT-qPCR for *STRA6*, *RBP1*, *CRABP1* and *RDH10*, using RNA from primary human fibroblasts, which had been treated with 1 µM ROL or 100 µM menadione for 24 h after 48 h of starvation in serum-free medium. N=2 cultures from 1 donor per treatment group.

Graphs show mean +/- S.D. *p<0.05, **p<0.01, ***p<0.001, ****p<0.0001 (One-way ANOVA, Šídák's multiple comparisons test (A, B), Two-way ANOVA, Tukey's multiple comparison test (C)).

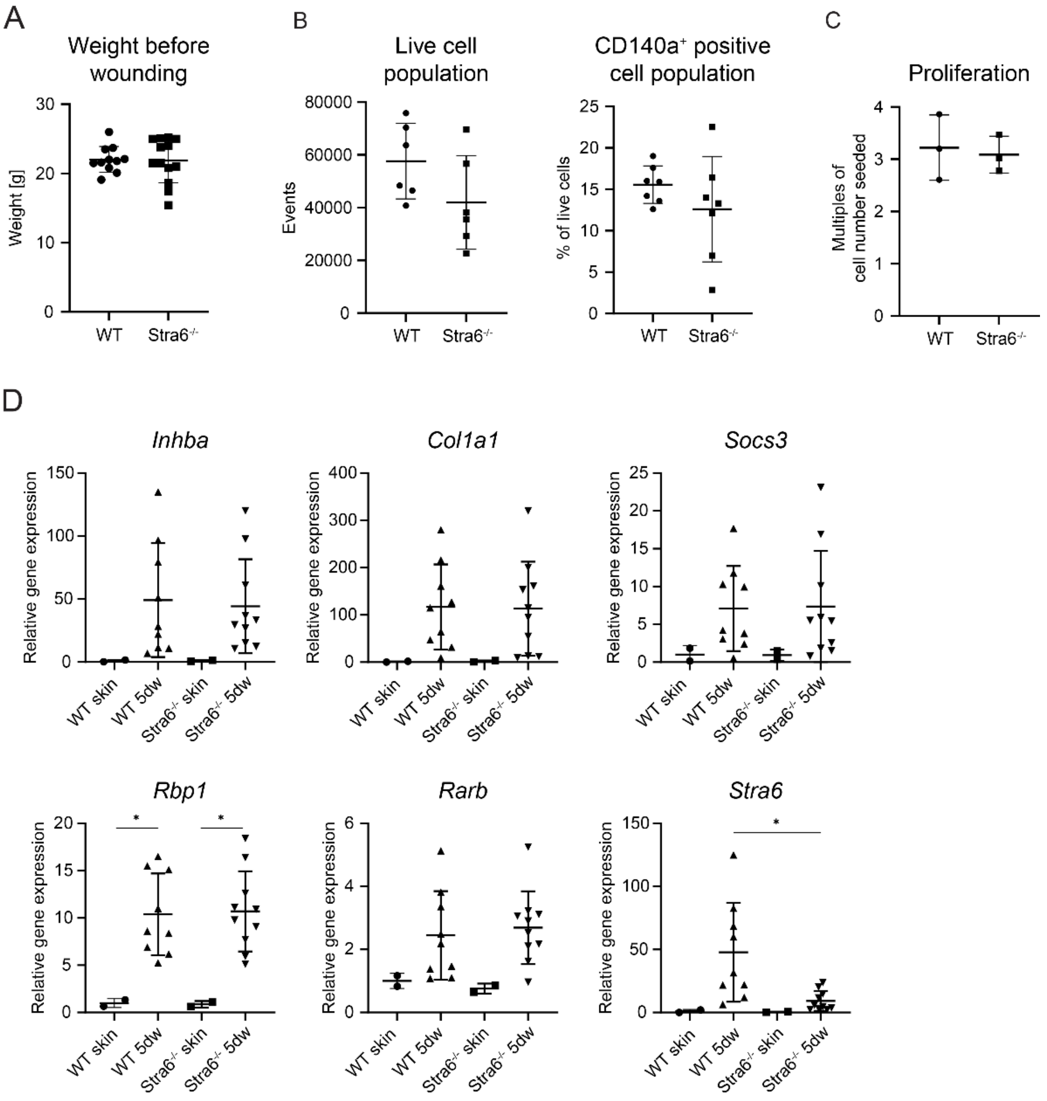

Supplemental Figure 3: *Stra6* knockout in mice does not significantly affect body weight or fibroblast numbers in the skin.

A. Body weight of WT and *Stra6*$^{-/-}$ mice before wounding. N=11-13 mice per genotype.

B. Total live cell population and percentage of Pdgfrα^{+} fibroblasts among all live cells in 5-day wounds of WT and *Stra6*$^{-/-}$ mice. N=7-8 mice per genotype.

C. Increase in the number of primary mouse fibroblasts from WT and *Stra6*$^{-/-}$ mice at 72 h after seeding of a defined number of cells and culturing in medium with 10% FBS. N=3 cultures from 3 mice per genotype.

D. RT-qPCR for *Inhba, Col1a1*, *Socs3*, *Rbp1, Rarb* and *Stra6* relative to *Rps29* using RNA from skin and 5-day wounds (5dw) of WT and *Stra6*$^{-/-}$ mice. N=2 (skin), N=9-10 (wounds) mice per genotype.

Graphs show mean +/- S.D. *p<0.05 (One-way ANOVA, Tukey's multiple comparisons test).

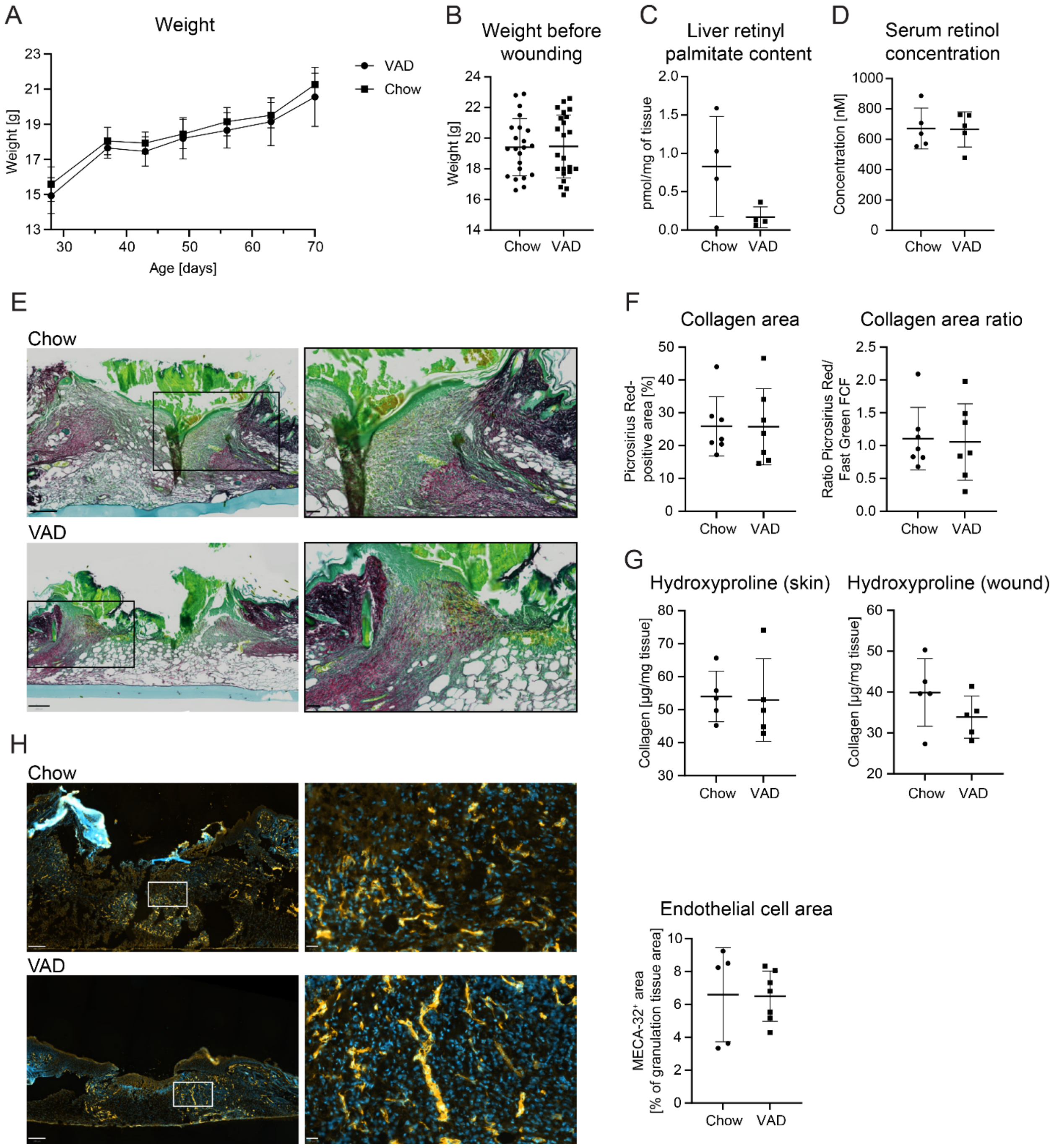

Supplemental Figure 4: VAD does not affect mouse body weight, collagen deposition in the granulation tissue and wound vascularization.

A. Body weight of chow-fed or VAD-treated mice in the weeks before wounding. N=7 mice per treatment group.

B. Body weight of chow-fed or VAD-treated mice on the day of wounding. N=22-23 mice per treatment group.

C. Liver retinyl palmitate content of chow-fed or VAD-treated mice measured by targeted metabolomics. N=4 mice per treatment group.

D. Serum retinol levels of chow-fed or VAD-treated mice after sacrifice measured by targeted metabolomics (day 5 after wounding). N=5 mice per treatment group.

E. Representative images of Picrosirius Red/Fast Green FCF-stained 5-day wound sections of chow-fed and VAD-treated mice. Picrosirius Red stains collagens (red), while Fast Green FCF stains areas of non-collagenous peptide (green).

F. Collagen analysis using Picrosirius Red/Fast Green FCF-stained wound sections. Intensity and area of the deconvoluted colors within the clot/granulation tissue were quantified; area represents percentage of collagen-positive tissue within the clot/granulation tissue. The area indicated with a black rectangle is shown at higher magnification at the right-hand side. N=7 mice per treatment group. Scale bars: 200 µm (overview) or 50 µm (detail).

G. Collagen analysis by quantification of hydroxyproline in acid-hydrolyzed skin and wound tissues. N=5 mice per treatment group (one wound per mouse).

H. Representative images of immunofluorescence stainings for MECA-32 (Cy3, red) of 5-day wound sections of chow-fed and VAD-treated mice. Hoechst was used to stain nuclei (blue). The area indicated with a white rectangle is shown at higher magnification at the right-hand side. The right panel shows the analysis of the MECA-32^{+} area in the granulation tissue of 5-day wound sections by quantifying the Cy3-positive area. The area is represented as a percentage of the granulation tissue. N=5-7 mice per treatment group. Scale bars: 200 µm (overview) and 20 µm (detail).

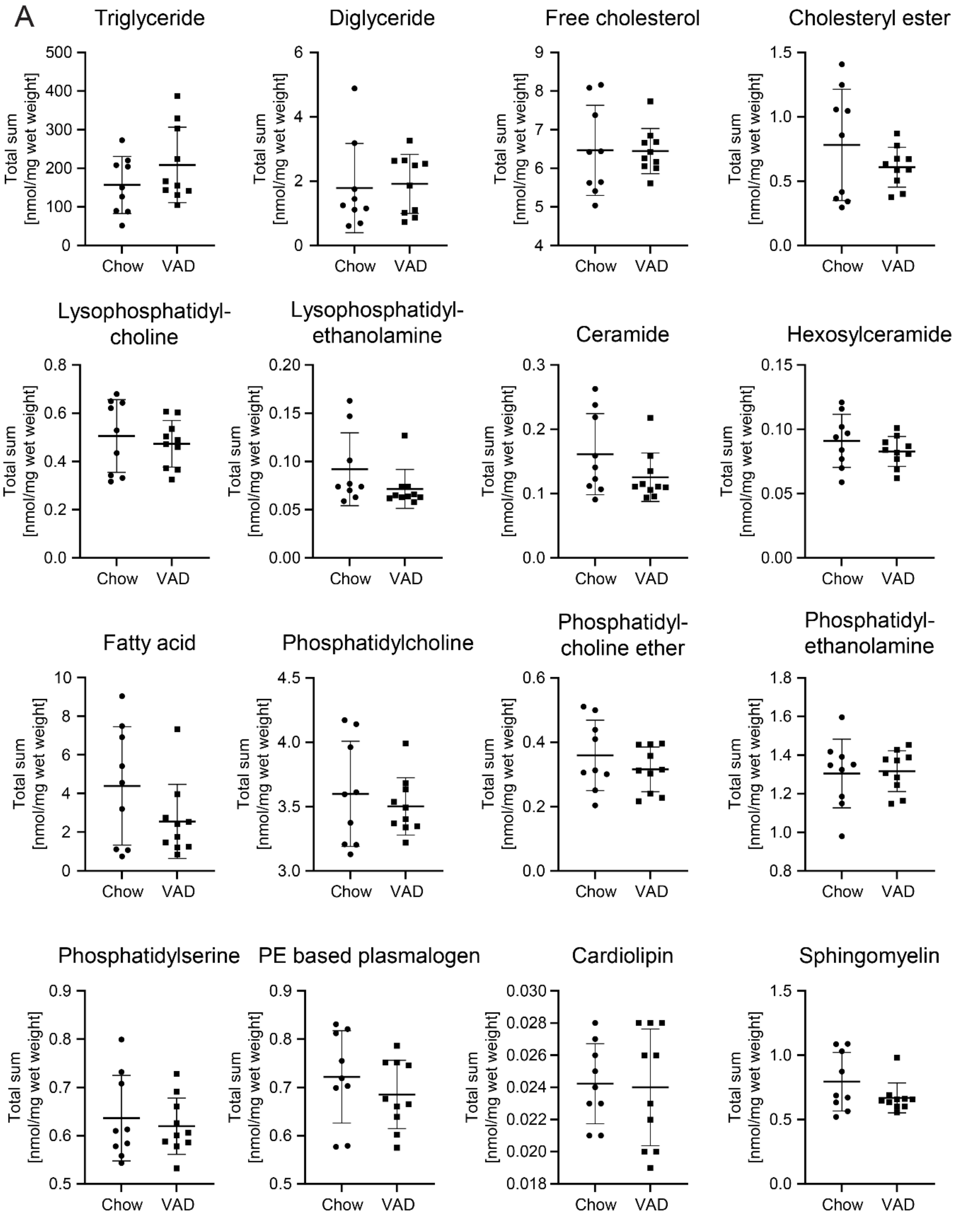

Supplemental Figure 5: VAD-treatment does not alter the lipid composition within the wound tissue (related to Figure 4).

A. Content of 16 different lipids in 5-day wounds of VAD- and chow-fed mice based on lipidomic analysis. N=9-10 mice per treatment group (one wound per mouse).

Graphs show mean +/- S.D.

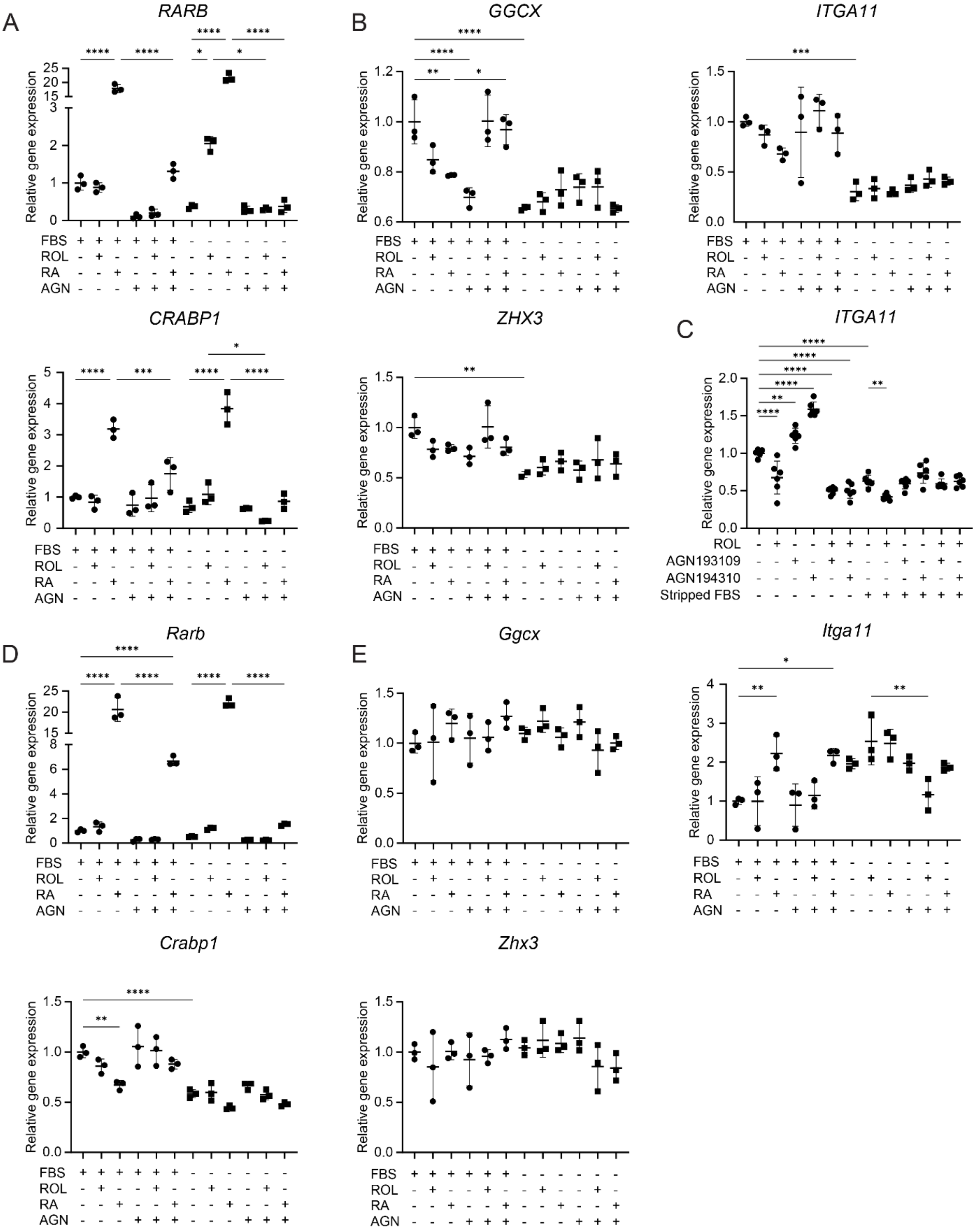

Supplemental Figure 6: *ZHX3*, *GGCx*, and *ITGA11* are not directly regulated by retinoids in fibroblasts.

A and **B.** RT-qPCR for *RARB*, *CRABP1* (A) or *GGCX*, *ITGA11*, and *ZHX3* (B) relative to *RPL27* using RNA from primary human fibroblasts, treated with 1 μM ROL, 0.1 μM RA, or 0.1 μM

pan-RAR inverse agonist AGN193109 (AGN) for 24 h, after 48 h incubation in medium with either 10% (+) or 0% (-) FBS. N=3 cultures from 1 donor per treatment group.

C. RT-qPCR for *ITGA11* relative to *RPL27* using RNA from primary human fibroblasts, treated with 1 μM ROL, 0.1 μM pan-RAR inverse agonist (AGN193109), 0.1 μM pan-RAR inhibitor (AGN194310), in the presence of charcoal-stripped FBS (Stripped FBS) or untreated FBS (for samples without Stripped FBS) for 24 h. N=6 cultures from one donor per treatment group.

D and **E.** RT-qPCR for *Rarb*, *Crabp1* (C), *Ggcx*, *Itga*11, and *Zhx3* (D) relative to *Rps29* using RNA from immortalized mouse fibroblasts, treated with 1 μM ROL, 0.1 μM RA, or 0.1 μM AGN193109 (AGN) for 24 h, after incubation for 48 h in medium with either 10% (+) or 1% (-) FBS. N=3 cultures from one mouse per treatment group.

Graphs show mean +/- S.D. *p<0.05, **p<0.01, ***p<0.001, ****p<0.0001 (One-way ANOVA, Šídák's multiple comparisons test).

Supplemental Tables and Legends

Supplemental Table 1: Regulation of genes associated with retinol metabolism in wound fibroblasts based on RNA-Seq data. RPKM: Reads Per Kilobase Million; FC: Fold Change; Bonferroni: Bonferroni corrected p-value.

Gene	Max RPKM	Log2 FC	Bonferroni
Rdh8	3.385	8.662	3.92E-06
Stra6	1.978	5.309	0
Lrat	0.061	*3.399*	*1*
Rdh10	22.764	2.233	0
Crabp1	96.217	1.987	8.43E-03
Rbp4	19.737	1.937	0
Rbp1	8.696	1.417	4.03E-02
Crabp2	11.067	*1.146*	*1*
Dhrs3	6.780	*0.689*	*1*
Dhrs4	9.043	*0.479*	*1*
Rdh11	8.103	*-0.451*	*1*
Aldh1a1	28.684	*-0.970*	*1*
Aox1	2.442	-1.039	3.52E-02
Dhrs9	8.389	*-1.203*	*1*
Awat2	0.809	-1.973	1.84E-02
Rdh12	2.406	*-2.037*	*1*
Adh7	21.979	-2.537	0
Cyp26b1	41.952	-2.653	9.82E-09
Ugt1a1	6.513	-2.878	1.53E-03
Sdr16c5	6.007	-3.726	1.99E-03
Rdh16	0.056	*-4.312*	*1*
Cyp1a1	0.966	-5.020	2.95E-04

Supplemental Table 2: Genotyping primers

Primer name		Sequence (5' - 3')
AGW277		ACTGACAGATCTATTCAGGAGGAGG
AHX114		AGGGGAGGATCCTTTCACCACC
HA418		CCTGCCCAAAGAAAGACTCA

Supplemental Table 3: Antibodies used for immunofluorescence staining, FACS or Western blot

Antibody	Cat.No.	Source	Dilution
Mouse anti-α-SMA	A2547	Sigma-Aldrich	1:1000 (WB)<br>1:400 (IF)
Rat anti-CD11b-BV711	101242	BioLegend	1:500
Rat anti-CD140a-APC	135908	BioLegend	1:200
Rat anti-CD45-Pacific blue	103126	BioLegend	1:400
Rat anti-F4/80-PE	123110	BioLegend	1:200
Rabbit anti-Ki67	ab15580	Abcam	1:200
Rat anti-MECA32	553849	BD Biosciences	1:1000
Goat anti-mouse Cy3	115-166-062	Jackson ImmunoResearch	1:200
Goat anti-mouse-HRP	W4021	Promega	1:5000
Rabbit anti-periostin	PA5-34641	Invitrogen	1:1000
Goat anti-rabbit-HRP	W4011	Promega	1:5000
Donkey anti-rat-Cy3	712-165-150	Jackson ImmunoResearch	1:200
Mouse anti-vinculin	v4505	Sigma-Aldrich	1:1000

Supplemental Table 4: Human RT-qPCR primers

Gene name	Sequence forward (5' - 3')	Sequence reverse (5' - 3')
CRABP1	CAAGTGCAGGAGTTTAGCCAC	CTCACGGGTCCAGTAGGTTT
GGCX	CCGCTTCCAGCAGAGGATTTT	TTGCCCAGGTCTTCACTCAC
ITGA11	TGTGATTCTGTGGTCCCGC	GAAATGGGGTGCCAGGAAGA
RARA	TCAGGCTACCACTATGGGGTC	CGGTCGTTTCTCACAGACTCCT
RARB	TGCCTGGACATCCTGATTCTTA	GGCAAAGGTGAACACAAGGTC
RARG	GCCCGAAAAAGTGGACAAGC	GCCCTTTCAGCTCCCTTAGTG
RBP1	CAACTGGCTCCAGTCACTCC	ACATTGACGTCGAGGGCG
RBP4	AACTTCGACAAGGCTCGCTT	GCACACGTCCCAGTTATTCA
RDH10	TCAACACGCAAAGCAACGAG	TCACCATTCCCAGCTTGCAG
RPL27	TCACCTAATGCCCACAAGGTA	CCACTTGTTCTTGCCTGTCTT
STRA6	TGACACACAGGACCAACCTTC	AGCCCAAGGCAGATAAAGGC
ZHX3	AATGGGGGAGGAGACCAGAG	CTGGGGACTCGATGTGTCAAA

Supplemental Table 5: Mouse RT-qPCR primers

Gene name	Sequence forward (5' - 3')	Sequence reverse (5' - 3')
Crabp1	CAAGTGCAGGAGTTTAGCCAC	CTCACGGGTCCAGTAGGTTT
Ggcx	CTCCCACCAGCACGTAAAGA	GAGGGTCAAAAAGCCTCTGC
Inhba	ACAGGTCACTGCCTTCCTTG	GGAGAACGGGTATGTGGAGA
Itga11	AGATACGCTGTGGCCGTTTT	TCCAGTCATAGGCTCCCACA
Lrat	TGGAACAACTGCGAACACTT	TAATCCCAAGACAGCCGAAG
Rarb	GAAAACGACGACCCAGCAAG	AATTACACGTTCGGCACCTTTC
Rbp1	GCCTTACGCAAAATCGCCAA	CCAGCTCACAGTGGTCATGC
Rdh10	AGGCTGCCGAATCAGAAAGA	CACACGACGGCTTCAAAAGG
Rps29	GGTCACCAGCAGCTCTACTG	GTCCAACTTAATGAAGCCTATGTCC
Socs3	ATGGTCACCCACAGCAAGTTT	TCCAGTAGAATCCGCTCTCCT
Stra6	GGACACACAGGGCCAATCTT	GGGATGTGGTCCCCAAGAAG
Zhx3	CAGAGGTGGTACGCTGGTTT	CAGGGGCTATGACCAGCAAA

Supplemental Table 6: LC method

LC System	Thermo Vanquish Horizon Binary Pump (Thermo Fisher Scientific)
Eluents	Solvent A: H_2O, formic acid (0.1%)<br>Solvent B: methanol, formic acid (0.1%)
Gradient	Linear, from 75% B to 98% B
LC runtime	8 min
LC column	Waters Premier BEH C18 column (50 mm x 2.1 mm)
Flow rate	1 ml/min
Column Temp	50 °C
Autosampler Temp	10 °C
Injection Volume	10 µl

Supplemental Table 7: MS method

Mass Spectrometer	Thermo TSQ Quantiva (Thermo Fisher Scientific)
Acquisition Mode	Selected reaction monitoring (SRM)
Q1 Resolution	0.7
Q3 Resolution	0.7
Fragmentation	CID fragmentation with argon (1.5 mTorr)

Supplemental Table 8: Peptide sequences for PRM analysis

Annotations	Peptide sequence
sp|Q9JI60|LRAT_MOUSE	SLLNEEVAR
sp|Q9JI60|LRAT_MOUSE	THFIHYGIYLGENR
sp|Q9JI60|LRAT_MOUSE	VAHLMPDILLALTNDK
sp|P09455|RET1_HUMAN	ALDVNVALR
sp|P09455|RET1_HUMAN	EIVQDGDHMIIR
sp|P09455|RET1_HUMAN	EFEEDLTGIDDR
sp|Q00724|RET4_MOUSE	YWGVASFLQR
sp|Q00724|RET4_MOUSE	FSGLWYAIAK
sp|Q00724|RET4_MOUSE	LQNLDGTCADSYSFVFSR
sp|Q9NYR8|RDH8_HUMAN	YQVVATMR
sp|Q9NYR8|RDH8_HUMAN	AVLPGMK
sp|Q9NYR8|RDH8_HUMAN	DLYLPASR
sp|Q8IZV5|RDH10_HUMAN	SVAGQVCLITGAGSGLGR
sp|Q8IZV5|RDH10_HUMAN	ENVYLTAER
sp|Q8IZV5|RDH10_HUMAN	FGVVGFHESLSHELK
sp|O14543|SOCS3_HUMAN	PLSSNVATLQHLCR
sp|O14543|SOCS3_HUMAN	IQCEGGSFSLQSDPR
sp|O14543|SOCS3_HUMAN	TVNGHLDSYEK
sp|O70491|STRA6_MOUSE	AASLDPGYHTYQNFLR
sp|O70491|STRA6_MOUSE	GAALDLDPPLQSIHPSR
sp|O70491|STRA6_MOUSE	TGAGSQGLQTSYSEK
sp|Q58HT5|AWAT1_HUMAN	DYFPITILK
sp|Q58HT5|AWAT1_HUMAN	TFPGITPHLATLSWFFK
sp|A2ADU9|AWAT1_MOUSE	NPSPEIVDK
sp|Q96PD7|DGAT2_HUMAN	YIGFAPCIFHGR
sp|Q96PD7|DGAT2_HUMAN	DTIDYLLSK
sp|Q96PD7|DGAT2_HUMAN	TLIAAYSGVLR
sp|O75911|DHRS3_HUMAN	VGDITILVNNAAVVHGK
sp|O75911|DHRS3_HUMAN	FSGTYTCMNTFK
sp|O75911|DHRS3_HUMAN	SLMDSDDDALLK
sp|Q8N3Y7|RDHE2_HUMAN	NVAGEIVLITGAGSGLGR
sp|Q8N3Y7|RDHE2_HUMAN	LLALQFAR
sp|Q8N3Y7|RDHE2_HUMAN	SFDVNFK
sp|O54754|AOXA_MOUSE	DGLTLGAGLSLDQVK
sp|O54754|AOXA_MOUSE	YIQDIVAATLK
sp|O54754|AOXA_MOUSE	LVLDEVTLAASAPGGK
sp|P00352|AL1A1_HUMAN	QAFQIGSPWR
sp|P00352|AL1A1_HUMAN	YCAGWADK
sp|P24549|AL1A1_MOUSE	IFINNEWHNSVSGK
sp|P29762|RABP1_HUMAN	QDGDQFYIK
sp|P29762|RABP1_HUMAN	VGEGFEEETVDGR
sp|P29762|RABP1_HUMAN	SSENFDELLK

sp|O43184|ADA12_HUMAN	LIEIANHVDK
sp|O43184|ADA12_HUMAN	FGFGGSTDSGPIR
sp|O43184|ADA12_HUMAN	VNSAGDPYGNCGK
sp|O14508|SOCS2_HUMAN	DSSHSDYLLTISVK
sp|O14508|SOCS2_HUMAN	EAPEGTFLIR
sp|O14508|SOCS2_HUMAN	TSAGPTNLR
sp|Q9NR63|CP26B_HUMAN	IFSHEALESYLPK
sp|Q9NR63|CP26B_HUMAN	ITLVPVLHPVDGLSVK
sp|Q9NR63|CP26B_HUMAN	DTHDTAPVFK
sp|Q9NQU5|PAK6_HUMAN	LSDFGFCAQISK
sp|Q9NQU5|PAK6_HUMAN	SDSILLTLDGR
sp|Q9NQU5|PAK6_HUMAN	LSVISSNTLR
sp|P10826|RARB_HUMAN	LPGFTGLTIADQITLLK
sp|P10826|RARB_HUMAN	LQEPLLEALK
sp|P10826|RARB_HUMAN	SSGYHYGVSACEGCK
sp|P13631|RARG_HUMAN	SSGYHYGVSSCEGCK
sp|P13631|RARG_HUMAN	LQEPLLEALR
sp|P13631|RARG_HUMAN	VQLDLGLWDK
sp|Q9BTZ2|DHRS4_HUMAN	TALLGLTK
sp|Q99LB2|DHRS4_MOUSE	FPSLGPYNVSK
sp|P10276|RARA_HUMAN	QLPGFTTLTIADQITLLK
sp|P10276|RARA_HUMAN	AHQETFPALCQLGK
sp|Q6PKH6|DR4L2_HUMAN/DHRS4_MOUSE	VALVTASTDGIGFAIAR

Supplemental Methods

Histology

Sectioning

Tissue samples were transferred into a histological cassette and fixed by o/n incubation in 4% paraformaldehyde (PFA) in PBS or acetic EtOH solution (95% ethanol and 1% acetic acid). Fixed samples were washed in PBS and then processed by stepwise dehydration using the Microm STP 120 Spin tissue processor (Thermo Fisher Scientific). Tissue blocks were sectioned, and sections (7 µm) were mounted onto SuperFrost Plus™ slides (Thermo Fisher Scientific) and dried at 37 °C o/n. The first section of each wound was marked for histomorphometrical analysis. Sections were dewaxed before staining using xylene (2×10 min) and rehydrated in a stepwise EtOH/H_2O gradient (100%, 96%, 80%, 70%, 50%, H_2O, 1 min each).

For cryo-embedding, tissue samples were submerged in tissue freezing medium (Leica Biosystems) in 15×15×5 mm Tissue-Tek® Cryomolds® and frozen on a metal block in liquid nitrogen. Cryosections (7-10 µm) were prepared, loaded onto SuperFrost Plus™ slides, and stored at -80 °C.

H&E staining

After dewaxing, paraffin slides were stained for 3 min with haematoxylin and 1 min with Eosin Y solution, air-dried, mounted with Eukitt® mounting medium (Sigma-Aldrich), and dried o/n at room temperature.

Herovici staining

After dewaxing and rehydration, sections were stained according to the following protocol:

- 5 min solution A (3.4 mg/ml Celestine blue, 42 mg/ml iron alum, 17% glycerol)
- 2 min running tap water
- 5 min solution B (2.5 mg/ml haematoxylin, 40.5 mM $FeSO_4$, 23.1 mM $FeCl_3$, 0.2% HCl, 25% EtOH)
- 2 min running tap water
- 2 min solution C (11 mM Metanil yellow, 1 drop/10 ml acetic acid glacial)
- 2 min solution D (0.45% acetic acid)
- 2 min running tap water
- 2 min solution E (0.2% saturated Li_2CO_3)
- 2 min solution F (0.45 mg/ml methyl blue, 6 mg/ml acid fuchsin, 9% glycerol, 0.045% saturated Li_2CO_3)
- 2 min solution G (1% acetic acid)
- 2 x1 min 100% EtOH
- 2x 10 min xylene

After staining, slides were air-dried, mounted with Eukitt® mounting medium, and dried o/n at room temperature.

Sirius Red/Fast Green staining

After dewaxing and rehydration, sections were stained in Fast Green solution (0.04% Fast Green FCF in saturated picric acid) for 15 min, then washed five times with distilled water and once for 20 s with water containing 0.5% acetic acid. After rinsing, sections were

stained in Fast Green/Sirius Red solution (0.01% Fast Green FCF and 0.04% Direct Red 80 in saturated picric acid) for 30 min, washed five times with distilled water and 1 min with acidified water, dehydrated with two 10 s washes in 95% ethanol and two 10 s washes in 100 % ethanol, and mounted with Eukitt®.

Preparation of charcoal-stripped FBS

FBS was incubated with 10 g/l dextran-covered activated charcoal under continuous rotation at 4 °C for 24 h. Charcoal was removed by filtration through a 0.45 μm filter, followed by sterile filtration through a 0.2 μm filter.

Treatments of cultured cells

Before most treatments, cells were starved for 48 h to deplete intracellular retinol stores. Starvation of primary human fibroblasts was done in serum-free medium, while primary and immortalized mouse fibroblasts were starved in medium supplemented with 1% FBS. Retinoids, AGN193109, and AGN194310 were dissolved in DMSO; control cells were treated with equal amounts of DMSO.

To examine the effect of retinol, primary human fibroblasts were treated with 1 μM retinol, 0.1 μM AGN193109, or 0.1 μM AGN194310 in medium either supplemented with 10% untreated (control) FBS or with 10% charcoal-stripped FBS without preceding starvation. To test retinol metabolism activation by retinoids, primary human or mouse fibroblasts were treated with 1 μM retinol (for both), 1 μM retinal, or 1 μM RA (for primary human fibroblasts only) for 24 h in the respective starvation medium. To stimulate primary human fibroblasts with an inflammatory cytokine, they were treated with 10 ng/ml TNF-α for 24 h in starvation medium. To induce ROS production in primary human fibroblasts,

they were treated with 100 µM menadione for 24 h after starvation. Titration of AGN193109 to find the optimal inhibitory concentration was done by treating primary human fibroblasts with 1 µM retinol and 0.1, 1, and 10 µM of AGN193109 in starved or FBS-supplemented medium, or by treating primary human fibroblasts with 1 µM retinol, 0.1 µM retinoic acid, and 10^{-3}, 10^{-2}, or 0.1 µM of AGN193109 in FBS-supplemented medium.

To analyze the expression of target genes of RAR signaling, cells were treated with 1 µM retinol, 0.1 µM RA, or 0.1 µM AGN193109 for 24 h in either starvation or normal medium.

RNA isolation from cultured cells

RNA isolation was performed with a Mini Total RNA kit (Qiagen) according to the manufacturer's instructions. Cells were harvested in 400 µl of RB buffer (included in the kit). RNA was eluted from the columns with 20 µl of RNase free water.

Cell culture

Primary cells

Human primary fibroblasts from foreskin tissue of two different donors and were kindly provided by Dr. Hans-Dietmar Beer (University Hospital Zurich, Zurich). The foreskin had been collected after informed written consent of the parents in the context of the Biobank project of the Department of Dermatology, University Hospital Zurich, and upon approval by the local and cantonal research ethics committee. They were obtained at passage 3 and used no later than passage 15.

Primary mouse fibroblasts were isolated from the dorsal skin of mice at postnatal day 2.5 (P2.5). To separate the dermal and epidermal layers, the skin was incubated in a 5% trypsin/EDTA solution for 1 h at 37 °C, after which the epidermis was gently removed. The remaining dermal tissue was finely minced and incubated with 2.5 ml collagenase type II solution (500 U/ml; Worthington Biochemical Corporation) for 1 h at 37 °C, with manual agitation every 15 min. The resulting cell suspension was filtered through a 100 µm cell strainer and centrifuged at 1200 rpm for 5 min. The cell pellet was resuspended in 8 ml of culture medium and distributed across two 6 cm culture dishes. The medium was replaced the next day, and cells were passaged before reaching confluency. The absence of mycoplasma contamination was confirmed using the PCR Mycoplasma Test Kit I/C (PromoCell).

Spontaneously immortalized mouse embryonic fibroblasts were kindly provided by Dr. Michael Cangkrama (ETH Zurich, Zurich).

Unless otherwise specified, all cells were cultured in high glucose DMEM supplemented with 10% FBS and 1% penicillin/streptomycin (P/S) in an incubator at 37 °C with 5% CO_2. Cells were passaged before reaching confluency. Experiments were conducted at 80-90% confluency. Cells were typically seeded at 50,000 cells/well in 12-well plates and at 100,000 cells/well in 6-well plates.

Analysis of primary mouse fibroblast proliferation

Freshly isolated primary mouse fibroblasts were seeded in 6-well plates at a concentration of 100,000 cells per well and cultured for 72 h before being detached and counted using a LUNA II™ automated cell counter.

Immunofluorescence (IF) staining

For IF staining of frozen sections, they were dried for 15 min at room temperature before washing twice with PBS. Fixation was done for 10 min in ice-cold acetone, followed by two PBS washing steps. Sections were permeabilized by three washes with PBS with 0.1% Triton X-100 and then rinsed with PBS twice. After marking the section with a hydrophobic pen, 12% BSA in PBS supplemented with 0.1% Tween-20 (PBST) was added for 1 h at room temperature to block unspecific binding sites. Next, 1:1000 anti-MECA-32 (BD Biosciences; #553849) antibody was added in blocking buffer and incubated for 48 h. Sections were washed thrice with PBST, and 1:200 anti-rat Cy3 (Jackson ImmunoResearch; #712-165-150) and 1:500 Hoechst were added in blocking buffer and incubated in the dark at room temperature for 30 min. After three washes with PBST, sections were coverslipped with Mowiol containing 10% DABCO.

For IF staining of acetic EtOH-fixed paraffin sections, slides were dewaxed, rehydrated, and washed for 5 min with PBS. They were permeabilized by 5 min washing with PBS with 0.1% Triton X-100 and rinsed with PBS. After marking the section with a hydrophobic pen, 5% BSA in PBS supplemented with 0.1% Triton X-100 was added for 1 h at room temperature to block unspecific binding sites. Next, 1:400 anti-α-SMA antibody (Sigma-Aldrich; #A2547) was added in blocking buffer and incubated o/n. After removal of the antibody, sections were washed thrice with PBST, and 1:200 anti-mouse Cy3 (Jackson ImmunoResearch; #115-166-062) and 1:500 Hoechst were added in blocking buffer and incubated in the dark at room temperature for 1 h. After three washes with PBST, sections were mounted with Mowiol containing 10% DABCO.

Imaging of tissue sections

Sections were imaged using a 3DHistech Pannoramic 250 slide scanner (3DHistech) or an Axioscan 7 slide scanner (Carl Zeiss AG) at 20x magnification.

Histomorphometry

For histomorphometric analysis, the first wound section cut from the center of the wound was used. Wound edges were defined by the last hair follicle, and measurements are specified in Figure 3C. Calculation of histomorphometric parameters was done in the following way:

$$Re-epithelialization\ [\%] = \left(1 - \frac{open\ wound\ length}{total\ wound\ length}\right) \times 100$$

$$Wound\ closure\ [\%] = \left(1 - \frac{open\ wound\ length}{initial\ wound\ length\ (5\ mm)}\right) \times 100$$

$$Contraction\ [\%] = \frac{total\ wound\ length}{initial\ wound\ length\ (5\ mm)} \times 100$$

$$Effective\ migration\ \left[\frac{\mu m}{\mu m}\right] = \frac{(total\ wound\ length - open\ wound\ length)}{sum\ of\ wound\ tongue\ lengths}$$

$$Neoepidermal\ thickness\ [\mu m] = \frac{sum\ of\ neoepidermal\ area}{sum\ of\ wound\ tongue\ lengths}$$

Semiquantitative scoring

H&E-stained sections were scored in a blinded fashion by two experienced researchers. Adipocyte area was scored from 0 (no adipocytes visible within the granulation tissue) to 3 (granulation tissue predominately consisting of adipocytes).

Hydroxyproline analysis of skin and wound tissue

Acid hydrolyzed samples of skin and wound tissue were analyzed using the QuickZyme Hydroxyproline Assay Kit as described by the manufacturer. Concentrations were inferred using a linear standard curve of a known collagen solution.

Serum collection

One ml blood was drawn from the left ventricle, left at room temperature for 15-30 min to coagulate and then centrifuged at 2,000×g for 10 min at 4 °C. The serum was frozen in liquid nitrogen.

26. Hu KH, Kuhn NF, Courau T, Tsui J, Samad B, Ha P, et al. Transcriptional space-time mapping identifies concerted immune and stromal cell patterns and gene programs in wound healing and cancer. *Cell Stem Cell*. 2023;30(6):885-903.e10.
