# Supplementary figures and images for: A multiomics analysis identifies retinol metabolism in fibroblasts as a key pathway in wound healing
Source: JCI Insight. 2025 Oct 16;10(22):e194188. doi: 10.1172/jci.insight.194188 (PMC12643503; doi:10.1172/jci.insight.194188)

## Full unedited blots for Figure 5E

Periostin

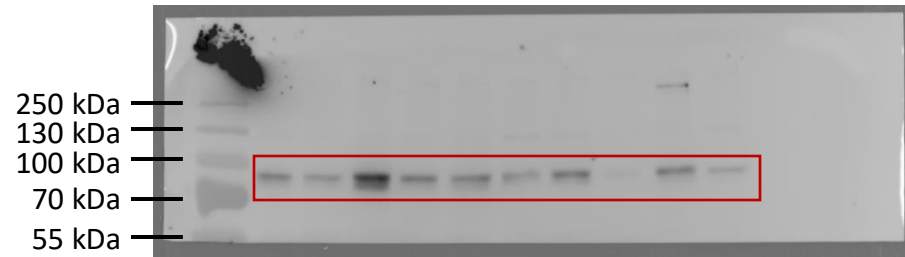

Vinculin

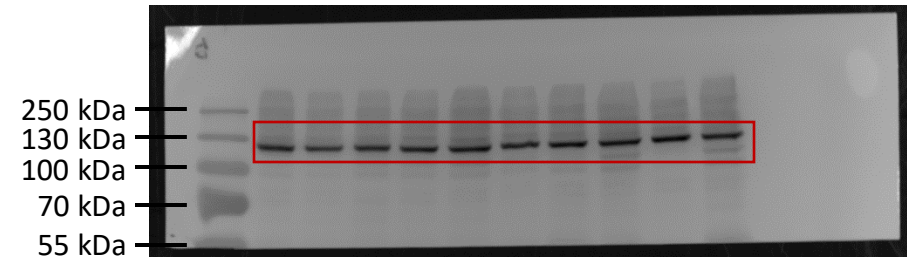

$\alpha$ -SMA

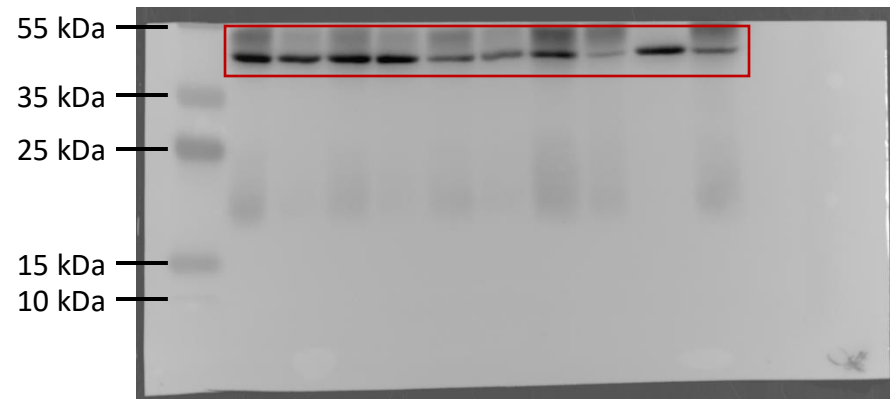

Supplement: Unedited blot and gel images [file jciinsight-10-194188-s178.pdf]
